# Social and occupational factors associated with psychological distress and disorder among disaster responders: a systematic review

**DOI:** 10.1186/s40359-016-0120-9

**Published:** 2016-04-26

**Authors:** Samantha K. Brooks, Rebecca Dunn, Richard Amlôt, Neil Greenberg, G. James Rubin

**Affiliations:** King’s College London, Department of Psychological Medicine, Cutcombe Road, London, SE5 9RJ UK; Public Health England, Emergency Response Department Science and Technology, Health Protection and Medical Directorate, Porton Down, Salisbury, Wilts SP4 0JG UK

**Keywords:** Disasters, Disaster response, Psychological impact, Systematic review, Wellbeing

## Abstract

**Background:**

When disasters occur, there are many different occupational groups involved in rescue, recovery and support efforts. This study aimed to conduct a systematic literature review to identify social and occupational factors affecting the psychological impact of disasters on responders.

**Methods:**

Four electronic literature databases (MEDLINE®, Embase, PsycINFO® and Web of Science) were searched and hand searches of reference lists were carried out. Papers were screened against specific inclusion criteria (e.g. published in peer-reviewed journal in English; included a quantitative measure of wellbeing; participants were disaster responders). Data was extracted from relevant papers and thematic analysis was used to develop a list of key factors affecting the wellbeing of disaster responders.

**Results:**

Eighteen thousand five papers were found and 111 included in the review. The psychological impact of disasters on responders appeared associated with pre-disaster factors (occupational factors; specialised training and preparedness; life events and health), during-disaster factors (exposure; duration on site and arrival time; emotional involvement; peri-traumatic distress/dissociation; role-related stressors; perceptions of safety, threat and risk; harm to self or close others; social support; professional support) and post-disaster factors (professional support; impact on life; life events; media; coping strategies).

**Conclusions:**

There are steps that can be taken at all stages of a disaster (before, during and after) which may minimise risks to responders and enhance resilience. Preparedness (for the demands of the role and the potential psychological impact) and support (particularly from the organisation) are essential. The findings of this review could potentially be used to develop training workshops for professionals involved in disaster response.

**Electronic supplementary material:**

The online version of this article (doi:10.1186/s40359-016-0120-9) contains supplementary material, which is available to authorized users.

## Background

Although there is a wealth of research on trauma-exposed populations, much of it has focused on individuals [1] rather than groups. This is unfortunate as people often experience trauma, particularly disasters (natural and human-initiated) together and intra-group processes may affect psychological outcomes. Some occupational groups may be unwillingly exposed to trauma, such as a commercial organisation targeted by terrorists, whilst others have a role in dealing with the aftermath of such events, such as emergency services personnel, disaster relief workers and healthcare staff who assist with an emergency response. With traumatic events becoming more prevalent across the world [2], it follows that organisations should consider their degree of disaster preparedness and possible impacts upon staff wellbeing and productivity [3].

In this systematic review we examined factors predicting psychological outcomes among any occupational groups who respond to disasters, in order to identify recommendations for interventions for reducing risk and fostering post-incident resilience in organisations. This study forms part of a wider review project on the impact of disasters on occupational groups. The current paper focuses on any employees responding to a disaster, while other papers within the same literature search explore disaster impact on ‘victim’ organisations [Brooks SK, Dunn R, Amlôt R, Greenberg N, Rubin GJ: Factors associated with psychological distress and disorder among occupational groups affected by disaster: A systematic review, in preparation] and healthcare workers responding to epidemics [Brooks SK, Dunn R, Amlôt R, Greenberg N, Rubin GJ: Factors associated with psychological distress in healthcare workers following an epidemic: A systematic review].

There have been previous reviews on the impact of particular disasters, such as the 9/11 terrorist attacks, on disaster responders [4] which have shown that post-traumatic stress disorder (PTSD) commonly affects such workers. There have also been reviews of factors affecting the mental health of particular groups of workers affected by disasters, such as humanitarian relief workers [5] and volunteers [6]. However to our knowledge this is the first review exploring the impact of all types of disasters – from natural through to human-initiated - on all types of responders, from emergency services personnel to social workers to nurses, on an international scale.

Our recent review of the impact of disasters on deployed humanitarian relief workers [5] identified many factors affecting psychological risk and resilience including: training; length and timing of deployment; traumatic exposure; emotional involvement; leadership; inter-agency co-operation; social and formal support; role; job demands; perception of safety; self-doubt; coping strategies; media exposure; and personal/professional growth. Whilst some of these may be specific to deployed relief workers, it is likely that others are generalisable to different occupations, such as emergency services personnel, social and healthcare workers, and those involved in disasters as victims rather than as responders. This review considered factors affecting both risk and resilience; that is, factors affecting wellbeing in either positive or negative ways. For example, perception of being in danger and lack of social support may be risk factors, or threats to wellbeing; conversely, perception of safety and adequate social support may facilitate resilience, i.e. be a ‘resource’. This approach can be related to Hobfoll’s Conservation of Resources model [7], which suggests that individuals accumulate and optimise resources which can be used to withstand threats. Resources may be personal (e.g. self esteem), organisational (e.g. role clarity), or task-related (e.g. receiving positive feedback). Experiencing a traumatic event can consume these resources, meaning there are not enough left over to withstand subsequent stressors, often resulting in burnout and stress. The model also suggests that some resources may enable individuals to secure further resources. Related to this model is the Job Demands-Resources model [8] which categorises working conditions as either demands (aspects of the job requiring effort/skills) or resources (aspects which help to achieve goals, or lessen demands).

Due to the explorative nature of this review we did not aim to test specific hypotheses. The main aim of the current paper was simply to answer the question: which social and occupational factors have been found to be associated with psychological wellbeing in disaster responders following a major incident?

## Methods

### Study selection

We included studies which were:

- primary, quantitative research;
- published in peer-reviewed journals;
- published in English;
- reported on social or occupational factors determining any outcomes related to psychological wellbeing in any occupational groups involved in responding to any disaster;
- and were published post-1984, 30 years before the study began in 2014; this also reduces the risk of including papers with data collected or analysed prior to the introduction of post-traumatic stress disorder as a diagnostic category in the DSM-III-R [9].

### Conducting the review

We composed a list of terms relevant to psychological wellbeing (Search 1). We used the Emergency Events Database (EM-DAT) [10] to assemble a list of extreme events (Search 2). We compiled a list of occupation-related terms (Search 3) and combined the three searches. The full strategy can be seen in Additional file 1.

In February 2015 one author (SKB) conducted the literature search using MEDLINE® (1946–2015), Embase (1980–2015), PsycINFO® (1806–2015) and Web of Science (1984–2015) databases. Resulting citations were downloaded to EndNote© software version X7, where duplicate citations were removed and titles were evaluated for relevance. Based on the inclusion criteria, two reviewers (SKB, RD) screened abstracts of the remaining citations to evaluate their relevance for the review and excluded any which were clearly irrelevant. Full-text copies of remaining citations were then obtained. SKB and RD read these papers in their entirety and excluded any not meeting inclusion criteria. Reference lists of key papers were searched for studies that may have been missed in the initial searches.

### Data extraction, quality appraisal and data synthesis

Details from relevant studies which were extracted included: year of publication; country of study; design; participants (‘n’ and demographic data); specific disaster; wellbeing outcomes and how they were measured; predictive factors and how they were measured; key results; conclusions; and limitations. Principal summary measures were measures of psychological wellbeing.

We assessed the quality of studies in three different areas: study design; data collection/methodology; and analysis/interpretation of results. Quality assessment forms were designed for a previous review [5] and informed by existing quality appraisal tools [11–13]. The appraisal tool can be seen in Additional file 2. Each study was given an overall score as a percentage, based on the number of ‘yes’ responses to the questions.

We used thematic analysis to group predictive factors into a typology. Topics we accepted as “themes” were required to be identified by at least two studies.

## Results

The initial search yielded 18,005 studies. 170 were deemed relevant to the wider study of occupational groups affected by disaster, and 111 of these related to responders and were thus accepted for inclusion in the current review. Details of the number of papers excluded at each stage of screening can be seen in Additional file 3. A summary of the papers identified can be seen in Additional file 4: Table S1. The majority (69) were cross-sectional. Almost half of the papers (50) focused on acts of terrorism, with 32 papers on the September 11th incident alone. Thirty eight papers focused on natural disasters (e.g. earthquakes, hurricanes), 22 papers looked at accidents (e.g. explosions, air crashes) and one paper looked at multiple incidents.

**Fig. 1:**
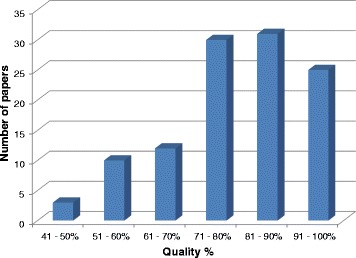
Scores for overall quality of included papers, assessed via quality appraisal tool

Overall quality, assessed as the total percentage of quality appraisal items endorsed for each study, was high (see Fig. 1) (mean % = 80.34 %; mode = 80 %, range 43.8–100 %).

Most studies scored highly for design; however common design errors included not stating the inclusion criteria or not recruiting participants during the same time period. Scores for method were mixed, with many studies reporting response rates of less than 50 % or not stating their response rate at all. Longitudinal studies often failed to give reasons for loss to follow-up. Most studies scored highly for the analysis and interpretation of results; those that did not generally failed to report confidence intervals or adjust for potential confounding variables.

Themes were grouped into pre-disaster, during-disaster and post-disaster factors. Each theme is discussed with examples from the literature. It should be noted that not every paper is discussed in the text, due to the large number of studies (111) included. Instead, we have summarised the findings for each theme and given examples to highlight these. All main findings have been discussed in the text, and any unusual results have also been reported. Full results of all 111 papers can be seen in Additional file 4: Table S2, and an overview can be seen in Additional file 4: Table S3 which summarises the number of studies with significant results for each theme.

### Pre-disaster

#### Occupational factors

Unsurprisingly, different occupational groups/professional levels respond differently to disaster. Several studies demonstrated significant differences in stress reactions between professional and non-professional (volunteer) responders. In several studies professionals had lower levels of post-traumatic stress disorder (PTSD), preoccupation and unpleasant thoughts [14–18], and found it easier to talk about their experiences than non-professionals [19] although one study found that professional fire-fighters had greater levels of PTSD than volunteers [20]. A small number of studies showed differences between occupational groups. For example, one study found differences in PTSD rates between different branches of the emergency services [21], another reported greater resilience in nurses than civilians [22] and another reported higher PTSD in health service staff who carried out domestic/home help duties than in medical staff [23].

Several studies found that longer employment acted as a protective factor, associated with lower stress, depression, burnout and PTSD [24–28]. However there were three studies which found that individuals with longer employment reported greater psychiatric and post-traumatic morbidity [29–31] and four further studies showing no significant association [32–35]. Chang et al. [29] suggest that rescue workers with more years of service are more likely to have had traumatic experiences (and perhaps residual symptoms from previous experiences). So, it may be that the conflicting results are due to previous work experiences: those with long employment and successful experiences may have positive outcomes, while those with long employment and experience of traumatic incidents or unsuccessful operations may have poor wellbeing.

General perceptions of one’s workplace and role pre-disaster also appeared to influence wellbeing outcomes post-disaster in a small number of studies. Low job satisfaction and lack of pride in the job were associated with PTSD in two studies [35, 36].

#### Specialised training and preparedness

Many studies found that provision of pre-disaster training and information enabled individuals to be emotionally and cognitively ready for the realities of what they may face, leading to better wellbeing outcomes [37–41]. Resulting from preparedness, confidence in one’s competence and knowledge appeared to impact post-disaster wellbeing. High sense of professional mastery and assurance in personal and team capabilities were found to reduce distress [37, 42, 43] while feeling that training had not prepared them well was associated with greater distress [21]. One study revealed no significant difference in distress between emergency care workers who had received training (related to psychological reactions to trauma) and those who had not [44]; however, rather than suggesting that training in general is not useful, the authors suggest that the training received was inadequate.

Evidence regarding the benefits of previous disaster experience was inconsistent. Some studies found prior experience was associated with greater distress [45, 46]. However several studies found no significant wellbeing differences between those who were involved in previous disasters and those who were not [14, 32, 33, 47–49] and one study found that previous experience was a protective factor [50]. It may be that the impact of previous disaster experience is mitigated by other factors: for example, one study [49] suggested that body handlers are a resilient group and have protective factors such as a strong sense of community.

#### Life events and health

Significant pre-disaster life events, including personal traumas and psychiatric history, were consistently found to be a risk for post-disaster mental health problems. Past mental health diagnoses increased the likelihood of reporting mental health symptoms post-disaster [38, 51–56]: it should be noted that many studies described this as ‘psychiatric history’ or ‘pre-existing psychopathology’ and did not describe which particular mental health diagnoses were reported. One study [31] found that previous psychiatric illness predicted anxiety but not significantly. Several studies found the risk of probable mental health problems to increase with increasing number of pre-disaster life events [23, 26, 30, 37, 47, 56–62]. It should be noted that while most studies specified that ‘negative life events’ or ‘adversity’ predicted poorer wellbeing, several studies simply reported on ‘prior life events’ without specifying whether these were adverse events. One study reported no significant differences between those with history of substance abuse and those without [63] while another [59] found that experiences during the disaster had a bigger impact on wellbeing than pre-disaster events. Two other studies showed no significant effect of previous trauma history [25, 49].

### During-disaster

#### Exposure

A substantial body of research has found that disaster exposure (in terms of severity and type of exposure) has multifaceted implications for psychological wellbeing. Many papers reported that traumatic exposure alone (irrespective of exposure type) predicted a range of psychological complaints and disorders, including anxiety, depression, general distress and PTSD [28, 33, 41, 44, 46, 50, 52, 55, 57, 64–71]. One study [72] found that disaster-exposed nurses had higher levels of PTSD, depression and psychosomatic symptoms during the disaster than non-exposed nurses, but lower psychosomatic symptoms after the disaster. Rates of distress were higher among those with repeated or high exposure [21, 24, 26, 56, 61, 73–78] and there was a dose–response relationship between the number of traumatic events experienced during a disaster and depression or PTSD [79, 80]. One study [48] found that exposure was correlated with distress but this was not significant in regression analysis, while four studies showed no significant effect of exposure on psychological wellbeing [49, 60, 81, 82]. Proximity to the epicentre of the disaster appeared to play an important role in psychological wellbeing [27, 83, 84]. With the exception of fire-fighters, rescuers responding to victims in the epicentre of a disaster appeared to suffer more PTSD symptoms than those farther out [18].

Dealing with serious injury or dead bodies appeared be a risk factor for psychological distress and post-traumatic stress responses. Workers with such exposure experienced stress, somatic complaints, fatigue symptoms, and were more likely to develop PTSD, depression, alcohol problems and anxiety [52, 56, 61, 64, 78, 83, 85–89]. Some research suggested that the type of exposure made a difference, with exposure to burns and child victims increasing the likelihood of PTSD [58]. Conversely, several studies did not demonstrate associations between exposure to bodies/injuries and mental ill-health [14, 33, 36, 90]. Again this inconsistency of evidence suggests there may be important mitigating factors making certain groups more resilient; one study [90] suggested that good ‘team spirit’ and morale may explain low levels of psychiatric morbidity in police body-handlers.

Few studies [44, 91] explored the relationship between disaster trauma exposure and positive outcomes, reporting that post-traumatic growth (PTG) was associated with higher levels of trauma exposure.

#### Duration on site and arrival time

Duration on site and number of hours spent in one shift generally appeared to be risk factors for mental ill health, although there was some inconsistency in the findings. Working long hours on the disaster site and not taking a day off each week significantly increased the risk of mental distress, job dissatisfaction and subjective health complaints [18, 36, 48, 54, 78, 92, 93] with increased likelihood in non-professional or non-traditional workers who may lack appropriate physical, mental and emotional preparation [56, 94]. Equally, prolonged time spent at a disaster site also significantly promoted distress. One study [95] found that the number of days spent on site was predictive of PTSD and depression, with evidence of more than 28 days [96], 90 days [64] and 120 days [97, 98] most significantly increasing the likelihood. However, some studies found evidence contrary to the above, with neither number of hours nor number of days being associated with psychological distress [17, 61, 79, 85, 99]. It may be the case that the participants in these studies were particularly resilient: for example, one [79] found that their participants were generally a resilient group with 81.0 % meeting the study’s criteria for ‘resilient’ (i.e. not meeting PTSD criteria at any of the study’s time points); similarly only a small percentage of participants in another study [61] met the criteria for full (as opposed to subsyndromal) PTSD suggesting they were particularly resilient.

Several studies found that earlier arrivals on the disaster site – i.e. being one of the first on the scene - were significantly associated with greater PTSD and depression [18, 61, 64]. The impact of the arrival time appeared quite specific. For example, arriving at the World Trade Center in the morning of 9/11 led to an increased risk for PTSD and depression that was significantly greater than even arriving in the afternoon of 9/11 [74]. Arrival in the afternoon was of a similar risk to arrival several days after the attack. Similarly, other studies [97, 98] found that the earliest of arrivals increased the likelihood of PTSD by as much as six times. One study [100] demonstrated that the prevalence of PTSD in the following 5–10 years was determined by time of arrival.

Conversely, several studies found no significant associations between arrival time and psychological distress post-disaster [17, 33, 47, 96]. This inconsistency in the literature may be due to many studies not controlling for training, preparation, equipment, or severity of disaster exposure: it is likely that those first on the scene will be less prepared, the evolving situation may be more ambiguous and they may be less well-equipped and going into a more dangerous environment than those arriving later. One study [56] found that (in non-traditional responders only) earlier arrival time was negatively correlated with PTSD. The authors acknowledge that this contradicts other research, and attribute it to the heterogeneous occupational composition of the sample and delayed traumatic exposure in workers without training who joined the recovery efforts late.

#### Emotional involvement

Several studies reported that employees identified with victims and became overly emotionally involved in the disaster. One study [27] found that stress increased along with the stress of the survivors being dealt with, while another [30] found that workers with a high level of identification with survivors had greater intrusive, obsessive and compulsive thoughts. Identification with victims as a ‘friend’ (i.e. envisaging the deceased as a friend; ‘this could have been my friend’), as oneself, or as a family member were associated with PTSD [101, 102].

#### Peri-traumatic distress/dissociation

Peri-traumatic dissociation during an incident increased the likelihood of acute stress disorder, PTSD and alcohol problems [28, 57, 63], while the number of dissociative symptoms further increased that likelihood [46]. High levels of peri-traumatic distress were associated with greater burnout and depression [51], psychiatric impairment [59] and PTSD [86, 103].

One study found no significant correlations between peri-traumatic dissociation and post-traumatic stress symptoms [104]; however it should be noted that this was based on a small sample (*n* = 25). A further study by the same authors [105] noted that rescue personnel experienced peri-traumatic dissociation but not any post-traumatic reactions, though they suggest that perhaps the post-traumatic response begins later, and suggest that the level of dissociation should be mapped from an early stage to predict whether it affects post-traumatic stress in the long term.

#### Role-related stressors

Work-related stressors were found to predict PTSD [26]. Role ambiguity and having insufficient job-related information were associated with increased anxiety, secondary traumatisation and job burnout [37, 78]. Being involved in tasks outside of usual remit, such as providing supervision when not in a leadership role and police officers fighting fires, increased the risk of PTSD [18, 97, 98]. Other studies found that working on damaged rooftops more than once [96] and fire-fighters performing construction duties [18] increased the probability of psychosomatic disorders and PTSD respectively. Furthermore, direct victim and local community contact substantially added to stress and distress. Certain tasks such as rejecting victims in need of help due to lack of resources or manpower, treating people who had been injured, cleaning up destroyed areas, handling residents’ complaints and being involved in crowd control were associated with PTSD and psychological distress [41, 44, 88, 89, 92]. Not being able to predict or control events, as well as feeling a lack of control over the nature and extent of victim injuries, were associated with post-traumatic stress in fire-fighters [42]. Other job-related predictors of poor mental health outcomes included: longer assignments, increased time with child clients, working with fire-fighters, and clients who discussed morbid material, for disaster mental health workers [25]; and qualitatively heavy workload for emergency service personnel [106].

Some studies reported no significant associations between job-related stressors and outcomes. For example, one study [30] found no association between high case load and psychological distress in social workers offering psychological support to disaster victims; another showed that high work demand was associated with increased alcohol and tobacco use in public health workers responding to hurricanes but not with PTSD or depression [76].

#### Perceptions of safety, threat and risk

Many papers showed a relationship between wellbeing and perceived safety (or risk) during the disaster. Low perceived safety (i.e. greater perceived risk to oneself) was associated with anxiety [107], depression [63], general psychiatric symptoms [21] and post-traumatic stress [42, 108, 109]. Subjective perception of danger to oneself was the single best predictor of PTSD in utility workers [52]. One study [78] found that worries about personal safety were predictive of PTSD, while feeling not enough safety measures were in place and concern about equipment quality were associated with anxiety.

Two studies reported non-significant findings regarding perceptions of personal safety, both by the same author and looking at fire-fighters. Perceived threat was significantly correlated with distress but did not remain significant after other factors were controlled for in regression analysis [48], while another study by the same author [81] found that volunteer fire-fighters with and without PTSD did not differ in terms of perceived threat.

#### Harm to self or close others

Having a near-death experience, being seriously injured or having a ‘severe mental trauma’ during the rescue predicted PTSD in rescue workers after an earthquake [83]: those who experienced one of these had a rate of PTSD 25.6 times higher than those who had not. Developing lower respiratory symptoms or skin rash were significantly associated with PTSD and depressive symptoms [94, 110]. Being injured predicted PTSD, depression, panic attacks and general anxiety [18, 43, 56, 109]. However, several studies showed no significant relationship between physical injury to the self and mental health outcomes [34, 48, 54, 60, 81].

Knowing someone injured or killed during the disaster was predictive of outcomes in many studies. Loss of someone close was associated with PTSD and distress [23, 44, 56, 61, 109]. Several studies also suggested that specific relationships (i.e. whether the person was a family member, colleague, friend or acquaintance) might predict outcomes differently. One study found an increase in PTSD risk for each additional death of a colleague [97] while another found that loss of a co-worker led to a near 4-fold increase in elevated PTSD and more than a 2-fold increase in use of a counselling service [75]. Having family members who died or were injured was associated with PTSD and depression [83, 88] and losing a family member appeared to have a greater impact than losing a friend [95]. Only one study [49] found no relationship between knowing anyone killed or injured and post-traumatic stress.

#### Social support

Many studies explored social support, generally finding that poor support was associated with reluctance to seek treatment [111]; PTSD, anxiety and depression [26, 28, 51, 64, 83, 112]; stress and illness [37, 69, 73]; secondary traumatisation and burnout [24]; and greater obsessive/compulsive and preoccupied symptoms [30, 113]. One study showed that general social support was not associated with either peri-traumatic dissociation or PTSD [114].

Several studies focused on organisational support in particular. Work culture support and supervisor support appeared associated with job satisfaction, work engagement, psychological strain and turnover intentions [115]. Conversely, poor relationships with line managers and co-workers predicted PTSD [36] and dissatisfaction with supervisory support was associated with depression [110], while poor workplace communication significantly increased the risk of mental distress [92]. High need for support and lack of organisational support in the disaster aftermath were the strongest contributors of depression in Red Cross volunteers [78]. However some studies found no significant associations between organisational support and outcomes [31, 33, 42, 61].

There were mixed results on the effect of friends/family support. Satisfaction with home support was not correlated with post-traumatic stress in one study [42] while family support was found to be protective in another [56]. Other studies found mixed results: for example, one study [116] found that social support from friends acted as a significant moderator on the relationship between trauma exposure and intrusion symptoms for UN soldiers but not for relief workers, while in another study number of sources of family support predicted full PTSD, but not subsyndromal PTSD [61].

Negative social behaviours were generally associated with poor wellbeing: being a target of harassment was associated with stress [41] and being assaulted (e.g. during crowd control activities) was a risk factor for PTSD in police [88].

### Post-disaster

#### Professional support

Though several studies examined whether employees felt immediate professional help (particularly debriefing) was helpful, only few examined whether receipt of professional help influenced mental health outcomes. There were mixed findings from those which did.

Not receiving psychological counselling during the rescue mission was predictive of PTSD in military responders following an earthquake [35] while Critical Incident Stress Debriefing (CISD) was found to help emergency medical workers cope [113]. Satisfaction with workplace debriefings was not associated with PTSD in fire-fighters; however, participants with other non-PTSD disorders were less likely to report satisfaction with the debriefings or recommend them to others [36]. Participation in a group counselling service was not associated with depressive symptoms [110]. One study [112] found that CISD led to higher avoidance, though this did not remain significant in multivariate analysis. Since so few studies explored the impact of debriefing on outcomes it is difficult to draw firm conclusions.

#### Impact on life

There were mixed results regarding the effect of having one’s personal life affected by the disaster. Having to spend nights away from one’s own home in the days following disaster did not predict PTSD in community volunteers after an earthquake [117], but needing food/water aid, clothes aid and financial assistance were predictive of PTSD, as was suffering financial difficulties due to the disaster. In a study of Red Cross volunteers [78], loss of their own resources (home, food, water, clothing or income) was the most influential exposure variable for depression. Another study [88] found that rare family contact and uninhabitable home were associated with depression. However, several studies showed that personal loss was not significantly associated with mental health outcomes [34, 48, 60, 81]. Losing one’s own property was a predictor of distress in several studies [48, 60, 92, 118] with only one study [81] finding no association between losses and PTSD.

Having one’s professional life affected by the disaster appeared to be predictive of wellbeing. Changes in the time and place of work, immersion in professional role and role expansion were correlated with post-traumatic growth [91]. Difficulty functioning at work post-disaster was associated with PTSD [97, 119] and acute stress [57], while job loss was also associated with PTSD [64, 67, 79]. Functional job impairment and taking mental health-related medical leave were associated with PTSD [53, 75].

#### Life events

Exposure to significant post-disaster life events (e.g. divorce, relationship break-up) was significantly associated with distress, PTSD, anxiety and depression [48, 60, 61, 89]. However in one study, exposure to subsequent fires did not influence mental health outcomes in volunteer fire-fighters [118].

#### Media

Watching television for 4+ hours per day, 1 month post-disaster, was predictive of PTSD symptoms in rescue workers [120] while another study [118] found that volunteer fire-fighters with persistent delayed-onset, persistent chronic and resolved chronic PTSD were all significantly more distressed by television reminders of the disaster. A third study also reported a positive correlation between anxiety and watching television [111]. Conversely, watching 3+ hours of daily media coverage was not associated with emotional distress in emergency care workers [44].

#### Coping strategies

Several studies explored the relationship between wellbeing and both positive and negative coping strategies. Most commonly, the studies considered avoidance or denial. ‘Avoidance coping’ , i.e. deliberate avoidance of traumatic thoughts, was associated with greater psychological distress [121, 122] and predicted traumatic stress [50]. Avoidant thoughts appeared to predict PTSD more strongly in fire-fighters with low exposure than intense exposure [81].

In terms of positive coping mechanisms, ‘proactive coping’ and positive thinking were associated with post-traumatic growth [27, 123] Another study [124] found that confrontive coping, distancing and planned problem-solving significantly reduced the effect of direct rescue effort involvement on general psychiatric morbidity.

Only one study found no significant relationship between coping strategies and outcomes [49].

## Discussion

We found evidence for a number of important risk factors which influence poor wellbeing in personnel involved in disaster response. Firstly, professional rescuers tended to fare better than volunteers, and individuals given roles outside of their expertise during the disaster tended to suffer more. This may be because professionals are more likely to be prepared; training and learned coping with daily work may explain lower psychological vulnerability in professionals. In particular those performing their regular roles during disasters are likely to have had more training and thus be more mentally and skilfully prepared. The small number of papers which compared outcomes across occupations tended to find that different occupational groups experienced different levels of PTSD. Future research is needed to establish which occupational groups are more at risk and why this might be; if certain occupations have different training or support needs, this should be considered when developing interventions to reduce the risk of mental health problems. We found inconsistent results regarding how duration of employment may affect outcomes. While further research may be useful, we suggest that sufficient preparedness is fundamental, and that care should be taken to train and prepare both new and long-term employees. Overall the literature suggests that there should be particular concern for the wellbeing of volunteers and those performing duties they have not performed before especially if they are ill-prepared.

Many studies demonstrated a significant association between exposure and outcomes; longer or more traumatic exposure generally was associated with poor outcomes – although in one study high exposure was associated with post-traumatic growth. There was inconsistent evidence that any specific exposure was especially traumatic, though most studies found that proximity to the epicentre of the disaster was associated with poorer wellbeing. Whilst any responder may be affected, those with the highest degree of exposure should be considered to be at especially high risk. We appreciate that due to lack of manpower and resources, it is likely to be difficult to reduce the long working hours of employees following disasters. However, it may be helpful to have rotating shifts, with employees able to take regular breaks. The literature also suggests it is important to ensure that particular care is taken to provide sufficient support to those first on the scene.

Role ambiguity was associated with poor outcomes, as were certain tasks, particularly those involving working with survivors and handling complaints from the public. Perceptions of high job stress during the disaster were generally associated with poor outcomes, though in many papers it was difficult to ascertain what was truly meant by ‘job stress’. Aspects appearing to be stressful included a perception of vulnerability or lack of control (e.g. lack of control over victims’ injuries or over one’s own tasks), unpredictability, and heavy workload. Due to the very nature of disasters, it is likely that there will be some loss of control and unpredictability involved. However, managers can work to mitigate the effects of this by, for example, ensuring role clarity, giving clear instructions, providing feedback and support, and preparing workers for the lack of control beforehand.

There were many papers suggesting that perceived risk and lack of safety were significantly associated with poor outcomes. Similarly, experiencing injury or a near-death experience appeared to predict poor wellbeing. Managers should ensure that employees are trained in safety measures beforehand and know which precautions to take, and also that all safety equipment is of adequate quality. This may minimise the amount of risk felt by employees. It may be particularly important to ensure that support is provided to those employees who suffer injury during the disaster, or who know someone injured or killed.

Social support appeared to be important, particularly organisational support, in terms of good relationships with leaders and co-workers. Managers should ensure that they are approachable and supportive, and establish camaraderie between co-workers (perhaps by sending workers on courses or workshops aimed at building team cohesion, and encouraging teamwork through the use of team-building exercises). Training employees in Psychological First Aid (PFA) or in Trauma Risk Management (TRiM) [125] may improve feelings of camaraderie while also training employees to support their peers. PFA training, providing a framework for supporting others following traumatic events, has been found to lead to greater confidence in supporting others’ psychological distress in Medical Reserve Corps members [126]. TRiM, which involves training in peer support, has been found to be useful in military personnel [127] and other trauma-exposed organisations such as railway employees and police [128, 129].

There were very little data on the effect of professional support (e.g. counselling, debriefing) on psychological wellbeing, and there were mixed findings from the few studies which did consider this, with some papers suggesting it was beneficial and others showing no effect. However, there were no papers showing a detrimental effect of professional help for disaster responders. More research is needed into the importance of support from professionals, and the best ways of providing such support (e.g. whether individually or to teams, and how quickly after the disaster it is likely to be helpful). Until then, it would be useful for organisations to be aware of the guidelines for managing traumatic stress [130, 131] and ensure that appropriate support is readily available for those who feel they need it; leaders should ensure that employees know what normal stress reactions after such an event may be, where to go to find help and that they feel comfortable in doing so.

Those whose personal or professional lives were affected appeared to be more at risk of mental health problems, with property loss and changes to employment strongly associated with outcomes. However, it should be noted that changes to professional life may well be a result of poor mental health, rather than a predictor: for example, someone with PTSD may perform poorly and lose their job. We recommend that organisations should consider the personal impact of disasters on their employees, and ensure support is available for those who suffer losses, particularly in terms of property or income. Traumatic post-disaster life events also appeared to negatively impact wellbeing, thus those who suffered traumatic events unrelated to the disaster should be considered a vulnerable group and supported appropriately.

Finally, research on coping strategies appeared to suggest that avoidance and denial were associated with poorer outcomes. This implies that acceptance of the situation, and being encouraged to face up to problems, may be helpful. Indeed, some studies showed that more proactive approaches were beneficial, such as confrontive coping. It may be helpful for employees to attend workshops encouraging coping strategies such as mindfulness; such training has been shown to have the potential to improve resilience in Marines [132], while brief workplace interventions including education about stress and training in relaxation techniques have been found to reduce symptoms of PTSD and anxiety trauma-exposed employees [133]. It has also been suggested that ‘approach’ coping (confrontive coping) and ‘avoidance’ coping (e.g. denial) can both be used effectively, depending on various situational characteristics: a study of police officers who took part in a coping skills programme designed to teach appropriate approach-avoidance coping strategies found that job-related stress was reduced and that this type of coping framework is effective for managing acute stress [134]. Further research on the effectiveness of coping styles within the context of a disaster would be useful.

Our review demonstrates that disaster responders are faced with a large number of demands which may be a threat to resources: being exposed to traumatic situations, spending long hours at the site, potentially becoming overly emotionally involved, having to perform tasks outside of one’s usual role, lack of control over events, feeling unsafe or in danger, potentially being injured or seeing others harmed or killed, and having one’s personal and professional life impacted post-disaster. Prior life events and mental health problems can exacerbate the effects of these demands. Our results show that appropriate training (leading to preparedness, a sense of personal competence, and belief in one’s own ability to perform their role) is an important resource. The other key resource appeared to be social support, particularly from colleagues, but also from family and friends. Some of the factors could be seen as either threats or resources depending on whether the individual in question was satisfied with the situation: for example, within our ‘perceptions of safety, threat and risk’ theme, it appeared that feeling in danger was a risk factor or threat while, for example, confidence in having adequate safety equipment and training in safety procedures could act as a protective factor or resource. Further research may consider looking in greater detail at the relationships between the various factors in order to develop a model which links them together.

### Strengths and limitations

The extensive list of all potentially relevant search terms and search of multiple high-quality databases, along with strict exclusion criteria and the rigorous screening process, add to the scientific merit of this review. The review was further strengthened by the number and variety of papers included, and the use of standardised data extraction spreadsheets to ensure that all papers underwent the same data extraction process. Finally, the use of a quality appraisal tool assessing all papers across several areas is an additional strength of the study, as we have been able to make clear (Additional file 4: Table S1, Additional file 4: Table S3) the quality of each individual study.

However the decision to limit the search to English-language papers may mean that important findings were missed; future reviews may consider comparing our results to foreign-language papers. The decision to include only papers published in peer-reviewed journals also presents a publication bias thus future studies may include grey literature. It also must be noted that there may have been selective reporting within the studies.

We identified many inconsistencies in the literature, with many high-quality papers presenting conflicting results about the same risk factor. Whilst study quality was generally high, most were cross-sectional studies and thus can only suggest associations rather than causality. Prospective, longitudinal studies and randomised controlled trials are needed to adequately assess risk factors. Additionally, many studies were retrospective; thus ‘pre-disaster’ risk factors were often measured post-disaster. Furthermore, studies often used vague terminology, for example ‘stress’, without defining what the term meant within the study and outcome measurement tools were highly varied making comparison of studies more challenging still. In spite of these limitations, this paper summarises key findings including potential interventions which may be useful to promote psychological resilience in responders.

### Implications

The recent United Kingdom Psychological Trauma Society guidance [131] for organisations whose staff work in high-risk environments suggests issues to think about and examples of both successful and unsuccessful interventions. Many of their recommendations fit with what was found in this review: for example, they emphasise preparedness by suggesting that new staff reflect on their suitability for the role before starting and that selection interviews should involve open discussion about the nature of the job. They also emphasise the importance of being prepared for the potential psychological impact of the job, proposing mental health training/briefings. Support is also highlighted, with suggestions for leadership and team training and peer support training programmes.

Based on the results of this review, we make the following recommendations for organisations with employees likely to be involved in disaster response:

- Organisations should have a clear policy framework on protecting staff within the management of a traumatic event.
- Managers should be aware of key risk factors and use these to identify particularly vulnerable groups who may need additional support.
- Employees should be trained in Psychological First Aid.
- All employees should receive appropriate and specialised training to equip them with the skills, knowledge and confidence to operate under challenging conditions.
- Workshops or training days on emotional/psychological wellbeing could be used to equip staff with knowledge and coping strategies.
- Organisations should provide opportunities for table top and/or varied simulated crisis training. Such exercises will draw together employees’ skills, knowledge and team cohesion to manage unpredictable events.

## Conclusions

Overall, we found many risk factors which could lead to poor wellbeing in disaster responders – for example, traumatic exposure, concerns about personal safety, bereavement – but it appears that the impact of these factors may be mitigated by appropriate training (and thus greater preparedness) and a good level of social support. Future research is needed to better understand predictors of resilience. However, from the literature examined in this review, it is possible to identify particularly vulnerable groups (e.g. those working in the epicentre of a disaster; those who arrived on the scene earliest; those with the greatest exposure; those performing tasks outside of their usual roles; those who were injured; those who experienced property or personal loss) and so it is particularly important that employees who fall into any of these groups are identified and adequately supported both during and after the disaster.

### Availability of data and materials

Not applicable.

## Additional files

Additional file 1: Search strategy – Search terms used in electronic databases. (DOCX 13 kb) Additional file 2: Quality appraisal – Quality appraisal tool. (DOCX 14 kb) Additional file 3: Flow chart – Screening and inclusion/exclusion. (DOCX 58 kb) Additional file 4: Table S1. Overview of literature. **Table S2.** Thematic analysis of literature. **Table S3.** Summary of results. (DOCX 111 kb)

## Abbreviations

EM-DAT: emergency events database
PFA: psychological first aid
PTG: post-traumatic growth
PTSD: post-traumatic stress disorder
TRiM: trauma risk management
