# Supplementary material for: Social and occupational factors associated with psychological distress and disorder among disaster responders: a systematic review
Source: BMC Psychol. 2016 Apr 26;4:18. doi: 10.1186/s40359-016-0120-9 (PMC4845476; doi:10.1186/s40359-016-0120-9)
Supplement: Additional file 1: — Search strategy – Search terms used in electronic databases. (DOCX 13 kb) [file 40359_2016_120_MOESM1_ESM.docx]

**Additional File 1 – Search strategy**

Search: EMBASE 1980 – 2015; Ovid Medline 1946 – 2015; PsycINFO 1806 – 2015; Web of Science 1984 – 2015.

Search 1 (psychological wellbeing);

Well?being; anxiety; panic; post?traumatic stress; PTSD; stress; “mental health”; depress*; neurosis; adjustment disorder*; distress; psychological; resilience; coping; “mental disorder*”; “positive psychology”; “satisfactory life”; mindfulness; flourish; pleasure; flow; growth

=COMBINE WITH OR

Search 2 (disasters);

Anthrax; avalanche; avian influenza; bioterrorism; bird flu; blizzard; bomb*; chemical spill; Chernobyl; cyclone; drought; disaster*; earthquake; Ebola; emergenc*; explosion; fire; Fukushima; H1N1; H5N1; hurricane; industrial accident; landslide; massacre; mass killing; MERs; Middle East respiratory syndrome; pandemic; nuclear radiation; radiological; SARs; severe acute respiratory syndrome; September 11^th^; shooting*; storm; swine flu; terroris*; Three Mile Island; tidal wave; tornado; tsunami; typhoon; volcanic eruption; volcano; World Trade Center.

=COMBINE WITH OR

Search 3 (occupational search terms);

Organisation*; organization*; occupation*; employee*; employer*; workforce*; worker*; business; team; emergency response; healthcare provider*; healthcare worker*; construction work*; fire?fighter*; fire officer*; paramedic*; doctor*; nurse*; police; first aid responder*; personnel; hospital administrator; military.

=COMBINE WITH OR

Combine Search 1 AND Search 2 AND Search 3
