# Supplementary material for: Social and occupational factors associated with psychological distress and disorder among disaster responders: a systematic review
Source: BMC Psychol. 2016 Apr 26;4:18. doi: 10.1186/s40359-016-0120-9 (PMC4845476; doi:10.1186/s40359-016-0120-9)
Supplement: Additional file 2: — Quality appraisal – Quality appraisal tool. (DOCX 14 kb) [file 40359_2016_120_MOESM2_ESM.docx]

**Additional File 2 – Quality appraisal**

All questions are answered with ‘yes’ or ‘no’.

*Section 1: Study design*

1. Was the research question/objective clearly stated?

2. Were all subjects selected or recruited from the same or similar populations (including the same time period)?

3. Were the inclusion and exclusion criteria for being in the study pre-specified and applied uniformly to all participants?

4. Was the study population and size clearly specified and defined?

*Section 2: Data collection and methodology*

5. Were standardised measures used, or where measures are designed for the study, attempts to ensure reliability and validity were made?

6. Were the data collected in a way that addressed the research issue?

7. Was the participation rate stated and at least 50%?

8. Was the number of participants described at each stage of the study?

9. If the study followed participants up, were reasons for loss to follow-up explained?

*Section 3: Analysis and interpretation of results*

10. Were details of statistical tests sufficiently rigorous and described?

11. Were details of confidence intervals given?

12. Were potential confounding variables measured and adjusted statistically for their impact on the relationship between exposure(s) and outcome(s)?

13. Was the answer to the study question provided?

14. Are the findings related back to previous research?

15. Do conclusions follow from the data reported?

16. Are conclusions accompanied by the appropriate caveats?
