# Supplementary material for: Social and occupational factors associated with psychological distress and disorder among disaster responders: a systematic review
Source: BMC Psychol. 2016 Apr 26;4:18. doi: 10.1186/s40359-016-0120-9 (PMC4845476; doi:10.1186/s40359-016-0120-9)
Supplement: Additional file 3: — Flow chart – Screening and inclusion/exclusion. (DOCX 58 kb) [file 40359_2016_120_MOESM3_ESM.docx]

**Additional File 3 – Flow chart**

Relevant for other reviews within this project but excluded for this paper
n = 59

Excluded after full-text screening
n = 401

Excluded after abstract screening
n = 3084

Excluded after title screening
n = 8167

Titles screened
n = 11,822

Studies included in review

n = 111

Full-text articles assessed for eligibility
n = 571

Abstracts screened
n = 3655

Duplicates removed
n = 6183

Total records found
n = 18,005

Additional records identified through other sources
n = 6

Records identified through database searching
n = 17,999

It should be noted that the search strategy was deliberately designed to be as broad as possible, to reduce the risk of missing potentially relevant papers, and over 18,000 papers were found in the initial search. We have therefore not included every reason for exclusion in this flow chart; however, some of the more common reasons for exclusion included lack of quantitative measures of wellbeing; measures of only physical and not psychological health; focus on preparedness for potential disasters as opposed to impact of real events; and failure to measure associations between outcomes and potential predictive factors.
