# Supplementary material for: Social and occupational factors associated with psychological distress and disorder among disaster responders: a systematic review
Source: BMC Psychol. 2016 Apr 26;4:18. doi: 10.1186/s40359-016-0120-9 (PMC4845476; doi:10.1186/s40359-016-0120-9)
Supplement: Additional file 4: Table S1. — Overview of literature. Table S2. Thematic analysis of literature. Table S3. Summary of results. (DOCX 111 kb) [file 40359_2016_120_MOESM4_ESM.docx]

Table S1. Overview of literature

| **Reference** | **Design** | **Participants (n)** | **Occupation** | **Disaster/crisis** | **Wellbeing outcomes** | **Time of measurement post-disaster** | **Quality appraisal score** |
| --- | --- | --- | --- | --- | --- | --- | --- |
| Adams et al., 2008 | Cross-sectional | 236 | Social Workers | September 11th | Compassion Fatigue Scale - Revised; GHQ-12 | 20 months | 86.7% |
| Alexander & Wells, 1991 | Comparison | 71 + 53 matched controls | Police | Piper Alpha oil rig explosion | Revised Impact of Events Scale; study modified Body Handling Questionnaire; Hospital Anxiety & Depression Scale; Eysenck Personality Questionnaire; Coping Strategy Scale | Pre & 3 months | 68.6% |
| Alvarez & Hunt, 2005 | Cross-sectional | 114 (82 deployed & 32 controls) | Canine search & rescue handlers | September 11th | PTSD Symptom Scale Self-Report; Beck Depression Inventory II; Beck Anxiety Inventory; Brief Symptom Inventory; Peritraumatic Dissociative Experiences Questionnaire; Structured Clinical Interview for DSM-IV; PTSD Symptom Scale Interview; Stanford Acute Stress Reaction Questionnaire | 6-7 months | 87.5% |
| Armagan et al., 2006 | Cross-sectional | 33 | Turkey Red Crescent Disaster Relief Team | Asian Tsunami | Clinician Administered PTSD Scale | 1 month | 93.3% |
| Bartone et al., 1989 | Longitudinal | 164 | Military Survivor Assistance Workers | Gander Military Air Disaster | Modified version of Personality Hardiness; study modified psychiatric health checklist; Bradburn Psychological Wellbeing checklist; Social Support Index taken from open-ended questions & coded. | 6 months & 1 year | 87.5% |
| Baum, 2014 | Cross-sectional | 63 | Social Workers | Gaza War | Brief Symptom Inventory; Impact of Events Scale; Secondary Trauma Scale; Post-Traumatic Growth Inventory | 6 months | 80% |
| Ben-Ezra et al., 2005 | Cross-sectional | 26 | Rescue Personnel | Hilton Hotel bombing in Sinai | Researcher administered Questionnaire incl - demographics; Impact of Events; Dissociative Experience Scale | 96 hours | 93.3% |
| Ben-Ezra et al., 2006a | Longitudinal | 25 | Rescue Personnel | Gas Pipe explosion in Tel Aviv | Researcher administered Questionnaire incl - demographics; Impact of Events; Dissociative Experience Scale | During & 36-48 hours | 80% |
| Ben-Ezra et al., 2008 | Cross-sectional | 23 | Emergency Rescue Personnel | Bet-Yehoshua Train Crash | Impact of Events Scale Revised; Dissociative Experience Scale; Centre for Epidemiologic Studies Depression Scale | 24 hours | 86.7% |
| Ben-Ezra et al., 2006b | Longitudinal | 5 | Rescue Personnel | Gas Pipe explosion in Tel Aviv | Researcher administered Questionnaire incl - demographics; Impact of Events; Dissociative Experience Scale | During & 36-48 hours | 60% |
| Ben-Ezra et al., 2013a | Cross-sectional | 73 | Hospital Personnel & civilians | Tohoku Earthquake & Tsunami, & Fukushima | Meaning of Life Questionnaire; Impact of Events Scale Revised; study-specific questions on coping, health & perceived threat. | 6 weeks | 53.3% |
| Ben-Ezra et al., 2013b | Repeated cross-sectional | 87 | Hospital Nurses | Gaza War | Impact of Events Scale Revised; Centre for Epidemiologic Studies Depression Scale; Psychosomatic Problems Scale | During & 6 months | 75% |
| Berninger et al., 2010a | Longitudinal | Total = 10,074  Year1 = 8,679 Year 2 = 1,161 Year 3 = 2,820 Year 4 = 4,166 | Fire-fighters | September 11th | Medical Monitoring Program - incl questionnaires on disaster exposure; physical health symptoms; mental health symptoms; and other variables. Elevated PTSD risk assessed in line with DSM-IV-TR. | Yearly, between 2001 & 2005 | 87.5% |
| Berninger et al., 2010b | Longitudinal | 5,656 | Fire-fighters | September 11th | PTSD Checklist-modified; individual questions on alcohol use and functional impairment. | With first 6 months & 3-4 years | 93.8% |
| Bhushan et al., 2012 | Cross-sectional | 20 | NGO Relief Workers | Indian Earthquake & Tsunami | Impact of Events Scale; Post-traumatic Growth Inventory; Dissociative Experiences Scale | 4 years | 73.3% |
| Biggs et al., 2010 | Cross-sectional | 90 | Rescue Personnel (police, fire-fighters, medics, search & rescue) | September 11th | Unnamed validated ASD measure; Zung Self-rated Depression Scale; SF-8 Health Survey; Peritraumatic Dissociation Experience Questionnaire | 2-3 weeks | 86.7% |
| Biggs et al., 2014 | Comparison | 1,623 | Police | Queensland Flood, Australia | General Health Questionnaire-12; study specific questions on disaster exposure | 10 months pre & 1 months post | 87.5% |
| Boscarino et al., 2004 | Cross-sectional | 236 | Social Workers | September 11th | Compassion fatigue scale-revised; Job-burnout scale; secondary trauma scale; GHQ-12 | 20 months | 80% |
| Bowler et al., 2012 | Longitudinal | 2940 | Police | September 11th | Post-traumatic checklist; study specific comorbidity questions | 2-3 years & 5-6 years | 87.5% |
| Brackbill et al., 2009 | Longitudinal | Wave 1 = 71,437  Wave 2 = 46, 322 | Adults incl rescue/recovery personnel | September 11^th^ | PTSD checklist | 2-3 years & 5-6 years | 87.5% |
| Brown et al., 2002 | Cross-sectional | 300 | Fire-fighters | Political violence in N. Ireland | GHQ-28 | During | 80% |
| Cardozo et al., 2005 | Cross-sectional | 589 | Local & expatriate aid workers | Kosovo | Harvard Trauma Questionnaire; Hopkins Symptom Checklist-25; General health Questionnaire-28 | Not reported | 93.3% |
| Cardozo et al., 2012 | Longitudinal | 19 NGOs= 211 pre-deployment; 169 post-deployment; 154 3-6 months post-deployment | Aid workers | Various – deployed to countries with widespread violence, chronic crises etc | Hopkins Symptom Checklist-25; Maslach Burnout Inventory-Human Services Survey | Pre-deployment; 3-6 months post-deployment | 93.8% |
| Cetin et al., 2005 | Cross-sectional | 434 + 154 controls | Military rescue workers | Turkey Earthquake | Impact of Events Scale | 3 months | 86.7% |
| Chang et al., 2003 | Cross-sectional | 84 | Fire-fighters | Taiwan Chi Chi Earthquake | Chinese Health Questionnaire; Impact of Events Scale | 5 months | 93.3% |
| Chang et al., 2008 | Cross-sectional | 193 | Fire-fighters | Taiwan Chi Chi Earthquake | Chinese Health Questionnaire (modified GHQ); Impact of Events Scale | 5 months post Taipei & 2 months post Taichung | 86.7% |
| Chiu et al., 2011 | Cross-sectional | 1,915 + 2,127 matched controls | Fire-fighters | September 11th | AUDIT, PTSD Checklist, Centre for Epidemiological Studies Depression Scale | 4-6 years | 93.3% |
| Corrigan et al., 2009 | Cohort study | 8,487 | Fire-fighters | September 11^th^ | Modified PTSD Checklist into binary form; counselling services unit data collection | During first 2.5 years | 93.3% |
| Creamer & Liddle, 2005 | Cross-sectional | 80 | Disaster mental health workers | September 11^th^ | Impact of Events Scale; Life Events Checklist; study specific questionnaire | 3-5 months | 86.7% |
| Cukor et al., 2011 | Mixed – Qualitative interviews & cross-sectional | 2,960 | Non-rescue / utility workers | September 11^th^ | Clinician Administered PTSD Scale (CAPS); Traumatic Events Interview; Structured Clinical Interview DSM-IV; Beck Depression Inventory; Brief Symptom Inventory; PTSD Checklist | 10-34 months | 80% |
| Dobashi et al., 2014 | Cross-sectional | 605 | Japan Ground Self-Defence Force | Great East Japan Earthquake | Japanese Impact of Events Scale - Revised; K10 - 1 month after disaster | 1 month | 93.3% |
| Durham et al., 1985 | Cross-sectional | 79 | Rescue workers | Apartment building explosion | Study specific questionnaire - 1 part adapted from Wilkinson (1983); another part adapted from Horowitz & Wilner's (1980) Coping Inventory | 5 months | 60% |
| Dyregrov et al., 1996 | Longitudinal | Time 1 = 57  Time 2 = 50 | Volunteer & professional disaster workers | Bus crash | Impact of Events; General Health Questionnaire | 1 & 13 months | 68.8% |
| Eidelson et al., 2003 | Cross-sectional | 592 | Psychologists | September 11^th^ | Study specific questionnaire | 3-5 months | 66.6% |
| Ehring et al., 2011 | Cross-sectional | 267 | Rehabilitation and reconstruction recovery workers | Northern Pakistan Earthquake | Impact of Events Scale-Revised; Pakistan Anxiety & Depression Questionnaire; Bradford Somatic Inventory (BSI); Maslach Burnout Inventory; Trauma Exposure Severity Scale; Past Traumatic Scale | 24 months | 86.7% |
| Epstein et al., 1998 | Longitudinal | 311 | Healthcare workers | Air show collision | Clinician administered structured clinical interview for DSMIII - non-patient; Impact of Events Scale; Symptom Checklist | 6, 12, 18 months | 50% |
| Ersland et al., 1989 | Cross-sectional | 134 | Professional & non-professional rescue workers | Alexander Kielland Oil rig disaster | Impact of Events Scale; Study specific questionnaire | 9 months | 80% |
| Evans, 2006 | Cross-sectional | 626 | Disaster Relief Workers | September 11^th^ | PTSD checklist; clinician administered PTSD scale; Brief symptom inventory; Beck depression inventory; State-Trait Anger expression inventory | 21-25 months | 53.3% |
| Evans et al., 2009 | Cross-sectional | 842 | Utility workers | September 11th | Clinician-administered PTSD Scale | 17-27 months | 73.3% |
| Fullerton et al., 2004 | Longitudinal | 628 | Rescue workers | United Airlines DC-10 plane crash | DSM PTSD-IV Scale; Zung Self-Rated Depression Scale | 2, 7, 13 months | 93.8% |
| Fullerton et al., 2013 | Cross-sectional | 2,249 | Public Health Workers | 2004 Florida Hurricane | PTSD Checklist; Patient Health Questionnaire-9 | 9 months | 93.3% |
| Gabriel et al., 2007 | Cross-sectional | 765 | Police, local residents & the injured | Madrid Bombings | Davidson Trauma Scale; Mini International Neuropsychiatric Interview-Spanish | 5-12 weeks | 80% |
| Grieger et al., 2003 | Cross-sectional | 382 | Military & civilian hospital staff | Virginia sniper attacks | Acute stress disorder inventory; CAGE questionnaire; PHQ-9; peri-traumatic dissociative experience questionnaire; Study specific perceived safety scale – 3 questions | 2-3 weeks | 86.7% |
| Gross et al., 2006 | Cross-sectional | 1,131 + 224 controls | Clean up & recovery workers | September 11^th^ | PTSD Checklist; PHQ-9 | 20 months | 66.6% |
| Guo et al., 2004 | Cross-sectional | 252 | Professional & non-professional rescue workers | Chi Chi Earthquake | Chinese Davidson Trauma Scale; Startle, Physiological, Arousal/Anger, Numbness – SPAN-C | 1 month | 73.3% |
| Hagh-Shenas et al., 2005 | Cross-sectional | 154 | Rescue workers – Red Cross, fire-fighters & volunteers | Bam Earthquake | General Health Questionnaire; Civilian Mississippi Scale for PTSD; Anxiety Sensitivity Index | 90 days | 60% |
| Hodgkinson & Shepherd, 1994 | Longitudinal | 67 | Social Workers | Piper Alpha North Sea Oil Explosion & Clapham Rail Crash | Social Readjustment Rating Scale; Hopkins Symptom Checklist; Psychological Wellbeing Scale; modified Personal Hardiness Scale; The following were asked in a study specific questionnaire with some questions formed from literature research – experiences; caseload; stress & Coping; Social Support; & Employment Role Issues. | Piper Alpha Sea Oil Explosion – 9 months & 12 months. Clapham Rail Crash – 4 months & 12 months | 68.8% |
| Holtz et al., 2002 | Cross-sectional | 70 | Human Rights Workers | Kosovo | General Health Questionnaire-28; Hopkins Symptom Checklist-25; Harvard Trauma Questionnaire (adapted for trauma events in Kosovo). | Not reported | 100% |
| Huang et al., 2013 | Cross-sectional | 923 (55 with PTSD + 868 without PTSD as control group | Military Rescue workers | Wenchuan Earthquake | Clinician Administered PTSD Scale during structured interviews; Eysenck Personality questionnaire; Trait Coping Style questionnaire; Social Support questionnaire; & self-report demographics questionnaire | 18 months | 87.5% |
| Huizink et al., 2006 | Cross-sectional | 1,996 | Police & Fire-fighters | Amsterdam Air Disaster | Self-rating Inventory for PTSD (SRIP); Symptom Checklist; International Classification of Primary Care (ICPC), as designated by the World Organization; Checklist Individual Strength; blood samples | 8.5 years | 100% |
| Jayasinghe et al., 2008 | Longitudinal | 1,040 | Disaster Relief workers | September 11^th^ | State-Trait Anger Expression Inventory; Clinician administered PTSD scale; Structured Clinical interview for DSM-IV; Brief symptom Inventory; Global severity index | Not reported – 1 year follow up of previous study in 2005 | 60% |
| Jenkins, 1996 | Mixed methods longitudinal | 36 | Emergency Medical workers | Mass shooting | Symptom Checklist-90-R; Study specific psychosomatic questionnaire | Retrospective pre, 8-10 days & 4 weeks post | 43.8% |
| Jenkins, 1997a | Longitudinal | 31 | Emergency Workers | Mass Shooting | Derogatis Symptom Checklist-90-R; study specific psychosomatic questionnaire | Retrospective pre, 8-10 days & 4 weeks post | 50% |
| Jenkins, 1997b | Cross-sectional | 68 | Fire-fighters & Police | Hurricane Andrew | Impact of Events Scale Health Questionnaire; Derogatis Brief Symptom Inventory | 2.5 months | 60% |
| Kaspersen et al., 2003 | Cross-sectional | 213 | UN Soldiers & Relief workers | Aid services & military services in Yugoslavia 1992-1996 | Trauma Exposure Questionnaire based on United Nations Interim Force In Lebanon study (Weisaeth, 1993); Post-Traumatic Stress Scale; Impact of Events Scale. | Not reported | 80% |
| Liao, et al 2002 | Cross-sectional | 836 | Rescue workers | Taiwan Earthquake | Brief Symptom Rating Scale | 2 months | 66.6% |
| Linley & Joseph, 2006 | Longitudinal | 56 at T1; 31 at T2 | Disaster Response Workers | Non-specific | Study-specific subjective appraisal scale; Impact of Events Scale; Post-Traumatic Growth Inventory; Changes in Outlook Questionnaire | Time 1 not reported; Time 2 = 6 months follow-up | 81.3% |
| Loganovsky et al., 2007 | Cross-sectional | 295 + 397 controls | Clean-up workers | Chernobyl | Russian & Ukrainian CIDI; IES; Symptom Checklist | 18 years | 86.7% |
| Long et al., 2007 | Cross-sectional | 5,448 | Red Cross workers | September 11^th^ | Impact of Events Scale – Revised | 12 months | 80% |
| Luce et al., 2002 | Cross-sectional | 1,064 | Health Service Staff | Omagh Bombing | PTSD Symptom Scale | 4-6 months | 73.3% |
| Luft et al., 2012 | Longitudinal | 20,841 | Police & non-traditional recovery workers | September 11th | PTSD Checklist | 3-7 years | 81.3% |
| Lundin & Bodegard, 1993 | Longitudinal | 50 | Various – interpreters, fire-fighters, dog handlers, teachers, medical staff & photographers | Armenian Earthquake | GHQ-28; Impact of Events Scale | Immediately post; 1 months post; 9 months post | 56.3% |
| Marmar et al., 1996 | Cross-sectional | Group 1 = 198 exposed professionals  Group 2 = 140 occupationally matched exposed civilians (controls)  Group 3 = 101 unexposed controls | Emergency Services Personnel | Interstate 880 Freeway collapse during the 1989 Loma Prieta earthquake in San Francisco | 8-item PDEQ-SV developed for this study; Impact of Event Scale-Revised; Mississippi Scale for Combat-Related PTSD adapted for EMS personnel; Social Adjustment Scale | Mean  Group 1 = 1.5 years  Group 2 = 3.3 years Group 3 = 4.1 years | 93.3% |
| Marmar et al., 1999 | Longitudinal FU of previous study | 322 | Emergency Services Personnel | Interstate 880 Freeway collapse during the 1989 Loma Prieta earthquake in San Francisco | Impact of Event Scale-Revised; Mississippi Scale revised for EMS workers; Symptom Checklist-90R; Social Adjustment Scale; The Peritraumatic Dissociative Experiences Questionnaire-Subject Version (PDEQ-SV) | Unclear  1.9years & between 3.3-5.1 years | 75% |
| Matsuoka et al., 2012 | Cross-sectional | 424 | Disaster Medical Assistance Team Workers | Great East Japan earthquake and tsunami which damaged the Fukushima Daiichi nuclear power plant | Kessler 6 Scale, CES-D, Peritraumatic Distress Inventory, IES-R. | 1 month | 80% |
| Mauer et al., 2007 | Cross-sectional | 1,423 | New York State Employees & New York State National Guard personnel | September 11^th^ | Medical Monitoring Evaluation | 11-19 months | 80% |
| McFarlane, 1987 | Cross-sectional | 469 + 162 comparison group | Volunteer Fire-fighters | Southern Australia Bushfires | Questionnaire designed for study comprising inventory of impact of disaster, a brief life events inventory, and the GHQ-12 | 4 months | 80% |
| McFarlane, 1988a | Longitudinal | 469 + 162 comparison group | Volunteer Fire-fighters | Southern Australia Bushfires | Questionnaire designed for study comprising inventory of impact of disaster, a brief life events inventory, and the GHQ-12 | 4 & 11 months | 86.7% |
| McFarlane, 1988b | Longitudinal | 469 + 50 at high risk of PTSD | Volunteer Fire-fighters | Southern Australia Bushfires | Structured interview developed for study; GHQ-12 | 4 & 8 months | 86.7% |
| McFarlane, 1988c | Longitudinal | 469 | Volunteer Fire-fighters | Southern Australia Bushfires | Questionnaire designed for study comprising inventory of impact of disaster, a brief life events inventory, and the GHQ-12; structured interview | 4, 8, 11 & 29 months | 87.5% |
| McFarlane & Papay, 1992 | Longitudinal | 469 | Volunteer Fire-fighters | Southern Australia Bushfires | Questionnaire designed for study comprising inventory of impact of disaster, a brief life events inventory, and the GHQ-12; structured interview (Diagnostic Interview Schedule) | 4, 11, 29 & 42 months | 87.5% |
| Misra et al., 2009 | Cross-sectional | 341 | London Ambulance Service Personnel | July 7^th^ London Bombings | Trauma Screening Questionnaire; list of 5 symptoms associated with adjustment disorders | 2 months | 80% |
| Morren et al., 2005 | Cross-sectional | 317 | Volunteer Fire-fighters | May 2000 firework depot explosion in Enschede | PTSD-srs & Symptoms Checklist | 3 years | 93.3% |
| Murphy et al., 2004 | Comparison | 73 | Fire-fighters | September 11^th^ | Impact of Events Scale | 6 months prior & 1 week post | 68.8% |
| Nishi et al., 2012 | Prospective Observational | 254 (173 at Follow-up) | Disaster Medical Assistance Teams | Great East Japan Earthquake | PDI; IES-R | 1 & 4 months | 93.8% |
| North et al., 2002a | Cross-sectional | 181 | Fire-fighters as rescue workers | Oklahoma City Bombing | Diagnostic Interview Schedule | Not reported (data collected spanned 27 months) | 68.8% |
| North et al., 2002b | Longitudinal | 181 | Fire-fighters as rescue workers | Oklahoma City Bombing | Diagnostic Interview Schedule | 34 months | 73.3% |
| North et al., 2011 | Longitudinal | 379 (228 at follow-up) | Office workers, utility and disaster response workers | September 11^th^ | Diagnostic Interview Schedule for DSM-IV | 3 & 6 years | 68.8% |
| Paton, 1994 | Cross-sectional | 37 | Fire-fighters & volunteer rescuer workers | Armenian Earthquake | Impact of Events scale together with short symptom checklist; 8 interviews | 3 months | 56.3% |
| Perrin et al., 2007 | Cross-sectional | 28,962 | Rescue & recovery workers – Police, Fire-fighters, Emergency Medical Services, Construction/engineering, sanitation workers, volunteer organisations | September 11^th^ | PTSD Checklist - Civilian | 2-3 years | 86.7% |
| Pietrzak et al., 2012 | Cross-sectional | 8,466 | Police | September 11^th^ | PCL-S; PHQ-9; PHQ; CAGE Questionnaire; Sheehan Disability Scale; study-specific questions about which services they think they might need in the next year | 0.8-7 years (mean of 4 years) | 86.7% |
| Pietrzak et al., 2014 | Longitudinal | 10,385 | Traditional (Police) & non-traditional responders (eg. Construction workers) | September 11^th^ | PCL-S | 3, 6, 8 years | 68.8% |
| Saroja et al., 1995 | Cross-sectional | 123 | Fire-fighters | Condominium collapse | Impact of Events Scale; GHQ (translated) | Within 1 month | 73.3% |
| Schwarzer et al., 2014 | Longitudinal | 2,943 | Police | September 11^th^ | PCL | ~2-3 years & ~5-6 years | 81.3% |
| Sloan et al., 1994 | Cross-sectional | 140 | Police, Fire-fighters, Medical & Mental Health Professionals | School Shooting | Impact of Event Scale (completed regarding how they felt in past week & also how they believed they felt immediately following the incident) | 6 months | 86.7% |
| Slottje et al., 2007 | Historical Cohort | 1,168 exposed + 828 unexposed | Police & Fire-fighters | 1992 Amsterdam Air Disaster | Dutch version of the 36-item Medical Outcome Study Short Form (SF36); Dutch version of the Self-Rating Inventory for Post-traumatic Stress Disorder | Mean of 8.5 years | 93.3% |
| Slottje et al., 2008 | Historical Cohort | 1,168 exposed + 828 unexposed | Police & Fire-fighters | 1992 Amsterdam Air Disaster | Dutch version of the Self-Rating Inventory for Post-traumatic Stress Disorder | Mean of 8.5 years | 93.3% |
| Soffer et al., 2011 | Cross-sectional | 20 | Rescue Personnel | Haiti Earthquake | Study-specific questions (1 item to measure each of life satisfaction, self-rated health and perceived coping); Hebrew version of the SPANE; Hebrew version of the SoMe; Peritraumatic Dissociative Experiences Questionnaire-Self Report Version; IES-R | 2 weeks | 80% |
| Soo et al., 2011 | Longitudinal | Year 1 = 8,669  Year 2 = 1,159  Year 3 = 2,816  Year 4 = 4,161  Year 6 = 4,343  Year 7 = 6,041  Year 8 = 6,588  Year 9 = 6,895 | Fire-fighters | September 11^th^ | PCL-C | Yearly, from 1 month to 9 years | 75% |
| Stellman et al., 2008 | Cross-sectional | 10,132 | WTC Office workers | September 11^th^ | PCL; PHQ; CAGE; Sheehan Disability Scale; single question | 10-61 months | 80% |
| Stewart et al., 2004 | Cross-sectional | 13 | Volunteer Responders | Swiss Air Flight-111 Disaster | Modified PTSD Symptoms Scale; COPE Questionnaire; Drinking Motives Questionnaire; info on quantity and frequency of drinking | 3 years | 73.3% |
| Suzuki et al., 2014 | Longitudinal | 3,743 | Public Servants | Great East Japan Earthquake | Kessler K6 Scale | 2 & 7 months | 93.3% |
| Tak et al., 2007 | Cross-sectional | 525 | Fire-fighters | Hurricane Katrina | CES-D short form | 13 weeks | 100% |
| Thoresen et al., 2009 | Cross-sectional | 581 | Various – NGOs, Police, Journalists, Government Officials, Travel agents, Medical & Health Personnel | South East Asia Tsunami | Impact of Event scale; 4 study specific questions | 9-10 months | 100% |
| Thormar et al., 2013 | Longitudinal | 506 | Red Cross Volunteers | 2006 Indonesian Earthquake | Impact of Event scale; HADS; SHC inventory scoring system | 6, 12 & 18 months | 81.3% |
| Thormar et al., 2014 | Longitudinal | 506 | Red Cross Volunteers | 2006 Indonesian Earthquake | IES-R; SHC Inventory; PDI; Sleep Quality subscale of the SCL-90R | 6, 12 & 18 months | 87.5% |
| Tosone et al., 2011 | Cross-sectional | 481 | Social Workers | September 11^th^ | 1 study-specific question; Connor-Davidson Resiliency Scale; Compassion Fatigue/Secondary Traumatic Stress subscale; PCL-C; STS was operationalised as the mean of scores on the PCL-C and compassion fatigue scale | 6 years | 86.7% |
| Tucker et al., 2002 | Longitudinal | 51 | Body handlers | 1995 Oklahoma City Bombing | DIS; Gallup interview; study-specific question | 2 years | 66.7% |
| Ursano et al., 1999 | Longitudinal | 54 | Volunteer body handlers | 1989 USS Iowa gun turret explosion | Impact of Event scale; SCL-90-R; DSMPTSD-IV Scale | 1, 4 & 13 months | 86.7% |
| Ursano et al., 2014 | Cross-sectional | 2,249 | Public Health Workers | 2004 Hurricane season in Florida | PCL-17 | 9 months | 100% |
| VanDevanter et al., 2014 | Cross-sectional | 362 | Nurses | Hospital evacuation due to Hurricane Sandy | Study specific questionnaire | 9-11 months | 53% |
| Valenti et al., 2014 | Longitudinal | 64 | Learning disability Support Workers | L’Aquilla Earthquake, | The Human Services Survey version of the Maslach Burnout Inventory | 1 month pre, 1 & 2 years post | 86.7% |
| Viel et al., 1997 | Cross-sectional | 1,412 | Chernobyl Liquidators | Chernobyl | ICD-9 diagnoses (unclear what disorders they looked at) | 8 years | 80% |
| Wang et al., 2010 | Cross-sectional | 343 | Healthcare Workers | Wenchuan Earthquake | IES-R | 3 months | 73.3% |
| Wang et al., 2011 | Cross-sectional | 1,056 | Military Personnel | Wenchuan Earthquake | Davidson Trauma Scale | 6 months | 93.3% |
| Warren et al., 2003 | Cross-sectional | 35 exposed + 73 unexposed | Emergency Medicine Physicians | September 11^th^ | Study specific (1 question) | 4 weeks | 66.7% |
| Weiss et al., 1995 | Cross-sectional | 367 | Emergency Services Personnel | 1989 Interstate-880 freeway collapse during San Francisco Bay Area earthquake | GSI of the SCL-90-R; IES-R and a version of the Mississippi Scale for Combat-Related PTSD modified for civilian EMS use | Not reported | 80% |
| West et al., 2008 | Cross-sectional | 912 | Police | Hurricane Katrina | PCL; CES-D | 8 weeks | 100% |
| Witteveen et al., 2007  Linked to Slottje et al., 2007 & 2008 | Cross-sectional | 1168 exposed + 828 unexposed | Police & Fire-fighters | 1992 Amsterdam Air Disaster | SCL-90-R; GHQ-12 (Dutch version); Self-Rating Inventory for Posttraumatic Stress Disorder; IES; Checklist Individual Strength | 8.5 years | 100% |
| Yokoyama et al., 2014 | Cross-sectional | 1,570 | Dispatched health Nurses | Great East Japan Earthquake | Unclear – possible study specific survey | 21-22 months | 80% |
| Zhen et al., 2012 | Cross-sectional | 210 + 236 controls | Red Cross Nurses | Wenchuan Earthquake | Traumatic Stress Symptom Checklist | Within 1 year | 80% |

Table S2. Thematic analysis of literature

| **Theme** | **Reference** | **Occupational group** | **Evidence** |
| --- | --- | --- | --- |
| PRE-DISASTER: Occupational factors (role, length of employment, job satisfaction) | Armagan et al., 2006 | Turkey Red Crescent Disaster Relief Team | *Length of employment:* No significant differences in PTSD caseness or CAPS-1 scores on basis of professional experience |
|  | Ben-Ezra et al., 2013a | Hospital personnel & civilians | *Role:* Nurses rated their health (t = 2.378, p<0.05) and life (t = 2.567, p< 0.05) as better, had higher perceived coping (t = 2.005, p<0.05), less fear of future disasters (t = 2.273, p<0.05), & presented lower PTSD symptoms (t = 2.622, p<0.05) than civilians.  Nurses appeared to have lower mental vulnerability and higher resilience - may be due to training & learned coping with daily work. |
|  | Boscarino et al., 2004 | Social Workers | *Length of employment:* Job burnout was negatively related to years working as a professional counsellor (b=-0.161, SE=0.095, Beta=-0.115, p<0.10), which is counter to expectations, although not significant. |
|  | Chang et al., 2003 | Fire-fighters | *Length of employment:* Multivariate logistic regression indicated that job experience (>3yrs; OR 23.05, 95% CI 3.39-156.85, p<0.001) was a significant predictor of psychiatric morbidity, and a significant predictor of posttraumatic morbidity (>3yrs; OR 6.87, 95% CI 1.52-31.14, p<0.05). |
|  | Creamer & Liddle, 2005 | Disaster mental health workers | *Length of employment:* Higher secondary traumatic stress was significantly positively associated with heavier prior trauma caseload (hours per week over pre 6months; r=0.33, p<0.05 & hours per week over career; r=0.32, p<0.05), & negatively associated with more years of professional experience (r=-0.23, p<0.05). |
|  | Dyregrov et al., 1996 | Disaster workers | *Role:* Professional status (volunteer or professional disaster worker) was significantly associated with level of difficulty felt in talking about experiences (X^2^=5.47, p<0.05), 75% volunteers & 43% professionals. |
|  | Ehring et al., 2011 | Recovery workers | *Length of employment:* Longer employment as a recovery worker was correlated with lower BSI scores (r = -0.16, p<0.05) |
|  | Eidelson et al., 2003 | Psychologists | *Length of employment:* Increased stress was significantly negatively associated with years of professional experience (β =-0.08, p<0.05) |
|  | Ersland et al., 1989 | Rescue workers | *Role:* 43% of non-professionals were left with strong emotional impressions, compared to 22% of professionals. Significant differences in stress reactions between non-professionals and professionals; uncertainty (p<0.001), anxiety (p<0.05), restlessness (p<0.001), hyperactivity (p<0.05). Significant relationship between poor mental health 9 months post & reporting severe/extreme uncertainty (p<0.001), restlessness (p<0.001), & apathy (p<0.01), and reporting mild/moderate irritation (p<0.05) & discouragement (p<0.01).  No significant differences between professional & non-professional rescuers regarding ability to cope (function as a leader, etc). |
|  | Guo et al., 2004 | Rescue workers | *Role:* Significant difference in PTSD prevalence using DTS-C between professional rescue workers (fire-fighters; 19.8%) and non-professionals (soldiers, 31.8%; p<0.05). The difference in the rate of PTSD using the SPAN-C between the professional rescuer group (21.6%) and the non-professional rescuer group (30.6%) was not statistically significant. |
|  | Hagh-Shenas et al., 2005 | Rescue workers – Fire-fighters, red crescent, student volunteers | *Role:* Significantly more student volunteers reached caseness for PTSD than red crescent or fire-fighters (X^2^(2, N=154)=18.02, p<0.01).  Student volunteers scored significantly higher for anxiety than the Red Crescent workers, t(45.51) = 3.85, p<0.01, and the fire-fighters, t(91.78) = 3.08, p<0.01.  Only the student volunteers scored greater than the fire-fighters on the depression subscale, t(118.35) = 5.00,p<0.01, but not the Red Crescent workers, t(116)= 1.96, p<0.05. |
|  | Hodgkinson & Shepherd, 1994 | Social Workers | *Length of employment:* The only significant finding in relation to professional experience was an unexpected tendency for the more experienced group to report greater symptomatology on the HSCL Anxiety subscale (t = -2.36, p<0.05). |
|  | Holtz et al., 2002 | Human Rights Workers | *Length of employment:* Those who had been working as a human rights worker for longer than 6 months, were at increased risk of developing anxiety (OR 4.8, p<0.05) and depression (OR 11.6, p<0.05). |
|  | Luce et al., 2002 | Health Service Staff | *Role:* Staff roles had significantly different PTSD scores F(7, 515)=4.07, p<0.01) - lowest for medical staff & highest for domestic/home help. |
|  | Marmar et al., 1996 | Emergency personnel | *Role:* The EMT/paramedic group showed significantly higher peri-traumatic dissociation than police; EMT/Paramedics and Caltrans groups were more symptomatic for PTSD than police; EMT/Paramedics had more social adjustment difficulties than police. Police reported more sick days than the Caltrans group.  Participants above the threshold for 'caseness' on the SCL-90-R were more likely to be EMT/Caltrans. |
|  | Misra et al., 2009 | London Ambulance Service personnel | *Length of employment:* Duration of employment was not associated with outcomes. |
|  | North et al., 2002a | Fire-fighters | *Job satisfaction:* PTSD was not associated with self-appraised work performance. Workers with PTSD less often acknowledged pride in their job (58% vs 86%, p<.01), had lower rates of job satisfaction (63% vs 81%, χ2=4.46, p<.05) and more often reported negative effects of the bombing on their job satisfaction (29% vs 10%, x2=7.15, p<.01).  Workers with post-disaster alcohol use disorder had lower self-reported satisfactory work performance (χ2=6.58, p<.05), were more likely to note negative effects of the bombing on their job satisfaction (24% vs 10%, p<.05) and had higher rates of functional interference due to PTSD symptoms (40% vs 22%, χ2=4.87, p<.05). |
|  | Paton, 1994 | Fire-fighters vs. volunteers | *Role:* Fire-fighters were more likely than volunteers to perceive staff problems, role uncertainty, leadership problems, access difficulties, not being able to do the job, inactivity, being pushed to the limit, and lack of support from other team members as stressors (p<0.05). Although not significant, fire-fighters were also more likely to report the magnitude of death and destruction, dealing with relatives, the numbers of dead, exhaustion, and equipment problems as prominent stressors. Volunteer group were more likely to perceive inter-team relations, communication problems and publicity as stressors (p<0.05). PTSD symptoms were more prevalent amongst fire-fighters (t=3.456, p<0.004). |
|  | Perrin et al., 2007 | Rescue and recovery workers (various) | *Role:* Compared to police, the highest PTSD prevalences were found among unaffiliated volunteers (adjusted odds ratio =3.7) and construction/engineering (adjusted OR =3.8). After controlling for significant demographic, disaster, and work experiences related to the World Trade Center, the prevalence of PTSD was substantially elevated in sanitation workers (adjusted OR=2.7) and individuals reporting affiliation with volunteer organizations (adjusted OR =2.0). |
|  | VanDevanter et al., 2014 | Nurses | *Length of employment:* There were no differences in reported deployment-related stress between new nurses and those with more experience. |
|  | Wang et al., 2011 | Military personnel | *Length of employment:* No differences in duration of military service between those with/without PTSD.  *Job satisfaction:* Dissatisfaction with current service (chi-square value=4.49, p=0.034) was significantly different between the PTSD/non-PTSD groups (PTSD group more likely to be dissatisfied). |
|  | Weiss et al., 1995 | Emergency services personnel | *Length of employment:* Years of EMS experience was weakly negatively related to general symptoms as measured by the GSI (r=-.10) and the summary measure of PTSD symptoms, the M-PTSD scale (r=-.13). It was not related to the experience of intrusive, avoidant, or arousal symptoms in the past week. |
| PRE-DISASTER: Specialised training and preparedness (training, sense of preparedness/competence, experience) | Adams et al., 2008 | Social Workers | *Sense of preparedness/competence:* Bivariate associations - Those who reported having enough information to perform their work effectively had lower secondary trauma (B=-0.14, SE=0.05, β = –0.19, p<.01). The higher respondents scored on the mastery measure, the lower their job burnout scores (B=-0.21, SE=0.05, β = –.26, p<.001). For the work information variable, having such information tended to lower job burnout but was non-significant (B= –0.10, SE=0.06, β =0.12, p<.10)  Multivariate associations - Those with higher social support and a higher sense of professional mastery were less distressed (B= –0.05, SE=0.02, β=-0.13, p<.05; B = –0.11, SE=0.03, β=-0.23, p<.001; respectively). |
|  | Alvarez & Hunt, 2005 | Canine Search & Rescue Handlers | *Training:* Federal Emergency Management Association certified handlers were less likely to report posttraumatic stress symptoms by self-report, *F*(1, 82) = −7.33, *p<*.05, *d*=−.70, and interview, *F*(1, 60)=−6.50,*p <*.05, *d*=−.62, than were non-FEMA handlers. |
|  | Armagan et al., 2006 | Turkey Red Crescent Disaster Relief Team | *Experience:* No sig differences in PTSD caseness or CAPS-1 scores on basis of previous traumatic event or previous disaster rescue. |
|  | Ben-Ezra et al., 2005 | Rescue personnel | *Experience:* Significant difference in post-traumatic reactions between those who experienced previous trauma and the novel exposure group (t = 2.692; p<0.05) - previous trauma group reported significantly less symptoms.  Significant differences in dissociative symptoms as well (t = 2.464, p<0.05), previous trauma group reported less dissociative symptoms. |
|  | Ben-Ezra et al., 2008 | Emergency Rescue Personnel | *Experience:* No significant differences between previously exposed & novel exposure groups in age, gender or perceived threat to life, or in IES scores. MANCOVA was conducted while controlling for age, gender and perceived threat to life, no significant differences found between exposure groups. |
|  | Fullerton et al., 2004 | Rescue workers | *Experience:* Those who were exposed with previous disaster experience were 6.77 times more likely to develop PTSD (CI 1.9-24.17, p<0.01). Even after adjusting for current disaster exposure, previous exposure remained a significant risk factor for PTSD (OR 6.34, CI 1.7-23.6, p<0.01). |
|  | Gabriel et al., 2007 | Police | *Training:* Apart from two police officers who reported symptoms consistent with depression, no other psychopathology was observed among the police officers. 70% of police officers interviewed were of the elite corps with extensive experience & training in handling terrorist attacks. |
|  | Liao et al., 2002 | Rescue workers | *Experience:* Previous disaster exposure had no significant predictive effect on psychological distress. |
|  | Lundin & Bodegard, 1991 | Various - Interpreters; fire-fighters; dog handlers; teachers; medical staff; photographer | *Training:* At 9 months FU clear difference in rate of questionnaire response between those who were educated & trained for rescue work & those who were not.  Trained professionals experienced significantly more unpleasant feelings during 1st week than non-professionals, but at 9 months this was reversed – no statistics presented.  Only 25% of all workers felt their training was sufficient - felt importance of disaster psychiatry, knowledge of possible personal reactions, theoretical knowledge & practical emergency skills.  Professionals were significantly less preoccupied by thoughts of being totally unprepared compared to non-professionals at all 3 post-disaster time points (1 week p<0.05 & 1 month p<0.001 & 9 months p<0.001). Those who felt unprepared were preoccupied by thoughts of the disaster.  At 9 months FU there was no difference in GHQ or IES scores between professionals & non-professionals. |
|  | Marmar et al., 1996 | Emergency personnel | *Training:* Participants above the threshold for 'caseness' on the SCL-90-R reported lower ratings of how well their training prepared them for the incident (p<.05). |
|  | McFarlane, 1987 | Volunteer fire-fighters | *Experience:* Previous fire experience was significantly correlated with GHQ score (p=0.006) but not with caseness on the GHQ (p=0.2) and was not significant in regression analysis. |
|  | Misra et al., 2009 | London Ambulance Service personnel | *Experience:* Previous exposure to a major incident was not associated with outcomes. |
|  | Murphy et al., 2004 | Fire-fighters | *Sense of preparedness/competence:* Five job threat variables were significantly correlated with IES score 6months pre-disaster: not being able to predict/control events (p<0.01), lack of control over nature/extent of victim injuries (p<0.001), **worries about personal competence in handling mass casualty runs (p<0.05),** exposure to increased personal risk (p<0.001) and concerns about personal injury/disability/death due to work (p<0.05). One week after 9/11 the same concerns were correlated but the magnitude of the relationships were slightly stronger especially for exposure to personal risk and concerns about personal injury/death. Associations were found with IES score and not being able to predict/control events (p<0.001), **worries regarding team competence when responding to disaster (p<0.001),** lack of control over victim injuries (p<0.001), **worries regarding personal competence in handling mass casualty runs (p<0.01),** exposure to increased personal risk (p<0.001), concerns about personal injury/death/disability (p<0.001). |
|  | Thoresen et al., 2009 | NGOs, police, journalists, health personnel, ministry of foreign affairs, travel agencies, chaplains, air force medics | *Training:* Specific preparation for the mission was associated with a lower level of stress reactions in disaster-area personnel (p<0.05). Training on work tasks was also associated with lower stress (p<0.05). |
|  | Tucker et al., 2002 | Body handlers | *Experience:* Prior professional disaster work was not significantly correlated with posttraumatic stress or seeking mental health treatment. |
|  | Ursano et al., 2014 | Public health workers | *Sense of preparedness/competence:* An increase in individual-level collective efficacy was associated with a significant decrease in PTSD severity.  Increase in community-level collective efficacy was associated with a significant decrease in PTSD severity.  In the full model, including all covariates, a one point increase in community-level collective efficacy was associated with a 0.22 point decrease (p,0.01) in PTSD symptom severity. The intra-communities correlation for the community-level efficacy model was 0.066 (Model x2=198.70, p,0.01).  Individual-level collective efficacy: Using a model that included all covariates, analyses revealed that **an increase in individual-level collective efficacy was associated with a decreased probability of having probable PTSD (OR=0.94, CI=0.92–0.97).** Further, having high injury/damage increased the probability of being diagnosed with probable PTSD (OR=2.63, CI=1.33–5.21).  Of those with high injury/damage, the probability of having PTSD is expected to be 0.12 if a person has a lower level of individual-level collective efficacy (score=20). This risk decreases sharply to 0.05 if he or she has a higher level individual-level collective efficacy (score=40), which is a 59% reduction. For those with lower injury/damage, the risk of having PTSD is expected to drop from 0.06 with a lower level of individual-level collective efficacy to 0.02 with a higher level of individual-level collective efficacy, which is a 67% reduction.  Community-level collective efficacy: In a model that contains all covariates, analyses revealed that an increase in community-level collective efficacy was associated with a decreased probability of having probable PTSD (OR=0.93, CI=0.88–0.98). Further, having high injury/damage increased the probability of being diagnosed with probable PTSD (OR=2.29, CI=1.19–4.39). |
|  | Warren et al., 2003 | Emergency medicine physicians | *Training:* Those who had received training and those who had not did not differ significantly in terms of distress (t(100)=1.29, p=.201). |
|  | Zhen et al., 2012 | Red Cross nurses | *Experience:* Traumatic stress symptoms were predicted by prior disaster experience. Earthquake-related fears were almost twice as common among first-time exposed nurses than those who had previously participated in rescue work. |
| PRE-DISASTER: Life events and health | Adams et al., 2008 | Social Workers | *Life events:* Those with more negative life events also had higher levels of psychological distress (B=0.04, p<.01), and higher job burnout scores (B =0.09, SE=0.03, β = 0.20, p < .01). |
|  | Alvarez et al., 2005 | Canine Search & Rescue Handlers | *Health:* Handlers with a past diagnosis of mental disorder were more likely to report symptoms of depression, PTSD, anxiety, and general psychological distress, (Fs(1, 60) > 10, ps < .05, ds> .67) and to meet criteria for a current mental disorder, (χ^2^(1, N = 60) = 5.64, p<0.05). |
|  | Biggs et al., 2010 | Range of rescue personnel | *Life events:* Disaster workers with probable ASD reported more pre-9/11 lifetime exposures to traumatic events than those without ASD (t=-2.51, df=87, p=0.014). The risk of probable ASD increased (OR = 1.35) with increasing numbers of reported pre-9⁄11 lifetime exposures to traumatic events (Wald X^2^= 5.43, p=0.020, CI=1.05– 1.73). Of the disaster workers who reported three or more lifetime traumatic events, 21.4% (N=12) had probable ASD compared to 3.0% (N = 1) of those with less than three traumatic events (X^2^ = 5.64, df=1, p=0.018). They were 8.73 times more likely to have probable ASD (Wald X^2^= 4.13, OR=8.73, p=0.042, CI=1.08–70.56). |
|  | Cardozo et al., 2012 | Humanitarian Aid Workers | *Health:* Previous mental illness contributed to anxiety 4.2 fold (CI 1.45-12.50). |
|  | Creamer & Liddle, 2005 | Disaster mental health workers | *Life events:* Own trauma history was not significantly associated with secondary traumatic stress. |
|  | Cukor et al., 2011 | Non-rescue / utility workers | *Health:* Risk of probable PTSD was 2.87 (p<0.001) times higher in those with psychiatric history, 3.47 (p<0.01) for those with psychiatric history and who felt their life was in danger.  Significant difference between vulnerability groups (no vulnerability, past trauma history, past psychiatric history, past both - F(3,2690)=95.97, p<0.001). No vulnerability & past trauma history was significantly different from all other groups, past psychiatric history was only diff from past both (all p<0.001).  51% of PTSD cases had neither past trauma nor psychiatric history.  Past trauma & psychiatric accounted for 8% of variance, exposure variables added another 8%. |
|  | Ehring et al., 2011 | Recovery workers | *Life events:* Symptom levels of PTSD were associated with past traumas (r = 0.39, p<0.001). |
|  | Epstein et al., 1998 | Military health workers | *Life events:* Increased risk of PTSD in number of stressful life events (OR 1.27; 1.15-1.41, p<0.001).  Individuals who developed PTSD at 6, 12, or 18 months experienced, on average, a significantly higher number of other stressful life events during the first 6 months after the disaster than subjects without PTSD (F=26.39, df=1, 296, p<0.0001). |
|  | Evans et al., 2009 | Disaster workers | *Life events / health:* Those with a history of trauma (t=-3.5973, p<0.0005), depression (t=-6.01, p<0.0001) or panic (t=-3.885, p<0.0005) reported significantly more social/occupational disability than those without psychiatric history. |
|  | Grieger et al., 2003 | Military & civilian hospital workers | *Health:* No associations found between previous substance abuse and acute stress disorder, peri-traumatic dissociation or depression. |
|  | Hodgkinson & Shepherd, 1994 | Social Workers | *Life events:* **39% of the variance in psychological wellbeing could be explained by** variables reflecting coping style (hardiness, p<0.01), **prior life events (p<0.001)** & role difficulties/contact with client's distress (identification p<0.05; 88% reported ruminating about client's experiences). |
|  | Holtz et al., 2002 | Human Rights Workers | *Health:* Those with previous psychiatric illness were at increased risk of developing anxiety (OR 1.6) and PTSD (OR 1.2), although not significantly. |
|  | Liao et al., 2002 | Rescue workers | *Life events:* Pre-disaster major life events (R^2^ = 0.03) predicted the severity of psychological distress. |
|  | Luce et al., 2002 | Health Service workers | *Life events:* Previous trauma associated with significantly higher PTSD scores (t(526)=3.05, p<0.01) – the type of trauma did not matter. |
|  | McFarlane, 1988a | Volunteer fire-fighters | *Life events:* To find whether the distress generated by the disaster (measured by the IES) similarly accounted for the association between threatening events which happened before the disaster and psychiatric impairment, the relationship between these variables was calculated. These variables were found to be significantly correlated (r = 0.17, P < 0.001). When the partial correlation of the number of life events before the fire with the GHQ score was calculated eliminating the effect of the IES, the relationship between the number of events and the GHQ score continued to be significant (GHQ x IES x previous life events; r = 0.15, P< 0.001). |
|  | McFarlane & Papay, 1992 | Volunteer fire-fighters | *Life events:* The group who had PTSD as well as some other disorder were more likely than the no disorder group to have experienced adversity both before the disaster (F(2127=4.2, p<.05) and after (F(2127)=6.0, p<.01). |
|  | North et al., 2002b | Fire-fighters | *Health:* The majority with any psychiatric disorder after the bombing (82%, N=56) had pre-existing psychopathology (versus 35% [N=40] of others) (McNemar χ2=15.07, df=1, p≤0.001). Excluding alcohol diagnoses, the rate of non-alcohol disorders after the bombing was four times higher in those with pre-disaster psychopathology (43%, N=21 of 49) than in those without pre-disaster psychopathology (11%, N=14 of 132) (McNemar χ2=4.67, df=1, p<0.05). Last, the rate of bombing-associated PTSD was significantly higher in those with a pre-disaster diagnosis of PTSD (38%, N=5 of 13) than in those with no pre-disaster diagnosis of PTSD (12%, N=19 of 168) (p<0.02, Fisher’s exact test). |
|  | North et al., 2011 | WTC employees | *Health:* 25 people without known qualifying exposures or who did not see the planes or the towers being attacked met PTSD symptom criteria. Of these individuals meeting symptom criteria without qualifying exposures, **20 (80%) had a pre-disaster psychiatric disorder** (compared with 25 of 48 [53%] of those with direct exposure to danger; χ2=4.74, df 1, p=.029). Of the remaining 5, 3 knew someone who was killed, 1 was 1.9 mi away, and 1 was 3.4 mi away but worked with families of 9/11 victims. |
|  | Pietrzak et al., 2012 | Police | *Life events:* Associated with full // sub-syndromal PTSD respectively:  Number of life stressors before 9/11 (1.55, 95% CI 1.45-1.66 // 1.27 (1.21-1.33) |
|  | Pietrzak et al., 2014 | WTC responders – including professional police responders & non-traditional responders such as construction workers | *Life events / health:* Police: those with a severe chronic, delayed-onset or recovering PTSD trajectory were more likely than those with a resistant trajectory to have been diagnosed with a psychiatric disorder pre-9/11 (p<0.001 for all) and report a greater number of stressors in the year before 9/11 (p<0.001 for all).  Non-traditional responders: Those with severe chronic, delayed-onset, sub-syndromal increasing, moderate chronic & recovering trajectory were more likely than those in the resistant trajectory to have been diagnosed with a psychiatric disorder pre-9/11 and report a greater number of stressors in the year prior. |
|  | Tosone et al., 2011 | Social workers in direct mental health practice | *Life events:* The path from experiencing prior traumatic events to secondary traumatic stress (STS) was significantly positive, suggesting that increased exposure to potentially traumatic life events is associated with STS. Enduring distress exhibited a positively signed and significant relationship to STS.  Significant indirect effects via resilience were observed for three of the four exogenous variables, namely avoidance (indirect effect = .03, p<.05), ambivalence (indirect effect = .03, p<.05), and **life events** (indirect effect = .02, p<.05). The magnitude of these indirect effects can perhaps be more easily expressed by considering the % of the total effect of each exogenous variable that is transmitted indirectly via resilience, the mediator. These %s ranged from 10-13% suggesting the effects of avoidant/ambivalent attachment and traumatic life events on shared trauma are primarily direct rather than indirect. |
|  | Tucker et al., 2002 | Body handlers | *Life events:* Personal experience with disaster and number of personal disasters were not correlated with seeking mental health treatment. |
| DURING DISASTER: Exposure | Alexander & Wells, 1991 | Police | Body handling and anxiety and depression revealed no sig associations.  No sig diffs found between body handlers and controls for sick days taken post-disaster. |
|  | Bartone et al., 1989 | Military - Survivor Assistance Workers | ANCOVA - dose response for psych wellbeing, wellbeing diminishes as a function of higher exposure. Main independent effects were found for exposure ((p<0.05), support (p< 0.05), and hardiness (P < 0.001). At high exposure levels, hardiness and support lend resilience. |
|  | Baum, 2014 | Social Workers | Stepwise multiple regression - Correlation with PTG, direct exposure alone explained 18% variance, PTSD symptomology & 2nd traumatisation added 15% & double exposure added 21% (personal & professional). |
|  | Ben-Ezra et al., 2008 | Emergency Rescue Personnel | No significant effects of exposure to dead bodies, most coped well. This might suggest that rescue personnel are more resilient to the sight of dead bodies than non-professionals are who happen to be in the vicinity. |
|  | Ben-Ezra et al., 2013b | Hospital nurses | During - Hospital nurses exposed to disaster (war) had significantly higher levels of PTSD symptoms (t = 3.22, p<0.01), psychosomatic symptoms (t = 2.501, p< 0.05) and depressive symptoms (t = 2.112, p<0.05), than nurses not exposed.  After - nurses in exposed group showed lower psychosomatic symptoms (t = -2.81, p<0.01).  MANCOVA revealed an interaction effect of exposure X time delay for somatization (F = 12.838, p<0.001), unexposed nurses after the war had high psychosomatic symptoms. Significantly higher mean depressive (t = 2.632, p< 0.05) and psychosomatic (t = 3.835, p<0.001) symptom levels in exposed nurses at time 1 compared to time 2. |
|  | Biggs et al., 2010 | Range of rescue personnel | Workers reporting one or more of the high-impact disaster exposures were 6.05 times more likely to have probable ASD compared to those with no high-impact exposures (Wald X^2^ = 6.60, OR = 6.05, p<0.010, CI = 1.53–23.88). |
|  | Boscarino et al., 2004 | Social Workers | Involvement in recovery efforts was positively related to secondary traumatisation (ST; b=0.166, SE=0.05, Beta=0.233, p<0.001). 52% of those with high recovery involvement were defined as a potential ST case, compared with 25% for those with low involvement (p<0.02). |
|  | Bowler et al., 2012 | Police | Significant difference in PCL scores for number of traumatic events witnessed at 9/11 (adjusted β= 2.17, adjusted SE=0.27, p<0.001), more events leading to higher PCL score. |
|  | Brackbill et al., 2009 | Rescue & office workers | Intense dust cloud exposure was associated with current PTS symptoms indicative of probable PTSD at Wave 2 - recovery workers: AOR 1.5 [95% CI, 1.3-1.6]; office workers: AOR, 1.7 [95% CI, 1.5-1.9].  Heavy layer of dust in the work place for office workers increased risk of PTS symptoms AOR, 2.0 [95% CI, 1.8-2.3])  Witnessing horror significantly increased PTS symptoms (AOR 1.3, 95% CI 1.2-1.5) in rescue workers. |
|  | Cardozo et al., 2005 | Aid workers | Dose response with the number of trauma events experienced during disaster significantly associated with depression; 1-3 events (AOR 2.6, CI 1.2-5.4) and 4+ events (AOR 5.3, CI 2.5-11.2) p<0.001 for expatriates & 4+ events (12.1, CI 2.3-66.7) p<0.01 for local workers. |
|  | Chiu et al., 2011 | Fire-fighters | Those who were captains & lieutenants (less exposure) were 0.7 (CI 0.6-0.9) times less likely to develop depression & 0.8 (CI 0.6-1.0) times less likely to develop PTSD compared to frontline fire-fighters with greater exposure. For fire chiefs, 0.6 (CI 0.4-1.0) time less likely to develop for depression and 0.5 (CI 0.3-0.9) times less likely for PTSD. |
|  | Corrigan et al., 2009 | Fire-fighters | Severe exposure group were 1.66 times more likely to use counselling services (CI 1.46-1.88, p<0.001). After adjusting for tenure, high PTSD scores & exposure exhibited a sig exposure response gradient (OR = 2.8 severe; 2.48 moderate; 2.22 mild) for attending counselling. |
|  | Cukor et al., 2011 | Non-rescue / utility workers | Extent of exposure predicted 89% of PTSD cases without psychiatric or trauma history (least vulnerable) but 67% of most vulnerable (both past psych & trauma).  Single best predictor was the subjective perception of danger to oneself, not actual occupational or personal exposure.  Risk of probable PTSD was 1.53 (p<0.01) times higher in those who saw body bags and 1.45 (p<0.05) times higher in those who saw body parts. |
|  | Dobashi et al., 2014 | Japan Ground Self-Defense Force Personnel | Dealing with dead bodies either as a primary role, or encountering them in another way, appeared be a strong significant (p<0.01) risk factor for psychological distress and post-traumatic stress responses when compared to workers without such exposure. |
|  | Durham et al., 1985 | Rescue workers | Rescue workers on the scene had significantly more symptoms than in-hospital workers (t = 2.44, p<0.02). |
|  | Eidelson et al., 2003 | Psychologists | When years of professional experience and gender were controlled for, distance from Ground Zero proved to be a significant predictor of responses on all survey items ( p<0.01), with psychologists working closest to the site of the attacks reporting the largest changes in professional and personal experience following 9/11. |
|  | Epstein et al., 1998 | Military health workers | Working at actual disaster site was not a significant risk for PTSD. Increased risk of PTSD in those who worked with burns patients (OR 2.88; 1.36-6.10, p<0.01); worked with child patients (OR 2.57; 1.32-5.01, p<0.01); and exposed to bodies from crash (OR 2.12; 1.07-3.16, p<0.05) |
|  | Fullerton et al., 2004 | Rescue workers | Exposed rescue workers had significantly higher rates of acute stress disorder (X^2^=82.1, p<0.001) than controls, & PTSD at 13months (X^2^=25.0, p<0.001) & depression at 7 & 13 months (p<0.05).  The likelihood of developing PTSD was 3.92 (CI 1.22–12.62) times increased if disaster exposure was high.  40.5% of exposed rescue worker compared to 20.4% unexposed had acute stress disorder, depression at 13months or PTSD. |
|  | Fullerton et al., 2013 | Public Health Workers | Workers with greater disaster exposure were more likely to have probable PTSD (OR=3.3) & depression (OR=3.6). After adjusting for demographics & work demand, those with high exposure were more likely to have probable PTSD (OR=3.21) & depression (OR=3.31). Those with high exposure had increased alcohol (OR=3.01) & tobacco use (OR=3.4).  The regression model, which included exposure, work demand, and the demographic variables, those with high exposure continued to be at increased risk of increased alcohol use for at least a 2-week period (OR= 3.09; X^2^=14.47, df=1, p<.001, 95% CI=1.78-5.38). |
|  | Gross et al., 2006 | Clean up & Recovery workers | Exposed workers with PTSD 3 times more likely to be exposed to human remains (CI1.87-4.82, p<0.0001), 2.62 times more likely to have witnessed death (CI 1.84-3.73, p<0.0001), 16.25 times more likely to have major depression (CI 11.07-23.85, p<0.0001) |
|  | Huang et al., 2013 | Military rescue workers | Rescuers at the epicentre suffered from PTSD symptoms 11.79 times that of rescuers who were not at the epicentre (OR = 11.79, 95%CI: 6.61–21.0).  Univariate analysis revealed that individuals who had contact with corpses, or witnessed the deceased or seriously injured suffered from PTSD symptoms at a rate 10.3 times higher than those without those experiences (OR = 10.35; 95%CI: 5.79–18.48). |
|  | Huizink et al., 2006 | Police Officers and Fire-fighters | Compared to unexposed police officers, exposed police officers had an increased likelihood of 2.8 (CI 1.5-5) for PTSD symptoms, 1.8 (CI 1.3-2.6) for high fatigue score, 2.1 (CI 1.5-2.8) for depression symptoms, 1.8 (CI1.4-2.3) for anxiety, 1.6 (CI1.3-2.0) for sleeping problems, 2.1 (CI 1.6-2.7) for somatic symptoms, obsessive-compulsive (all p<0.05). |
|  | Loganovsky et al., 2007 | Clean-up workers | Since 1986, clean-up workers were 1.7 times more likely to have a depressive disorder than controls (CI 1.0-2.7, p<0.05), were 4.0 times more likely to have an anxiety disorder than controls (CI 1.3-12.5, p<0.05) & were 2.1 times more likely to experience suicidal ideation compared to controls (CI 1.1-4.1, p<0.05).  **Level or amount** of exposure to disaster (high vs. moderate/low) had significant effects on mean score on somatization (p<0.05), avoidance (p<0.001), hyperarousal (p<0.01), and intrusion (p<0.005) subscales and overall PTSD symptoms (p<0.001), with high exposure workers scoring higher. |
|  | Long et al., 2007 | Red Cross Workers | Significant differences were found among both function (direct & indirect roles; F(4, 2284) = 3.64, P<0.025) and exposure groups (F(4, 2460) = 5.36, P<0.001) on dependent measures. Direct services and exposed groups were found to have higher scores on the outcome measures than their counterparts; the amount of the difference in scores was not meaningful. |
|  | Luce et al., 2002 | Health service workers | Those involved in some way had significantly higher PTSD scores than those not involved (t(1062)=-11.09, p<0.001). Capacity of involvement significantly affected PTSD score F(2, 533)=18.34, p<0.001). Involvement in both professional and civilian capacity increased PTSD scores compared with purely professional involvement (p<0.001). |
|  | Marmar et al., 1996 | Emergency personnel | Participants above the threshold for ‘caseness’ on the SCL-90-R reported greater critical incident exposure (p<.01). |
|  | Marmar et al., 1999 | Emergency personnel | Higher exposure was associated with higher stress response (p<0.001 for intrusion, avoidance & hyper-arousal). |
|  | Mauer et al., 2007 | New York State employees and NYS National Guard personnel | Participants caught in the 9/11 cloud of dust were more likely to report some psychological symptoms, including feeling jumpy/easily startled (PR (prevalence ratio) 2.40, CI 1.42-4.05), concentration problems (PR 1.85, CI 1.20-2.85), emotional numbness (PR 1.83, CI 1.16-2.90), flashbacks (PR 1.76, CI 1.02-2.46), and difficulty remembering things (PR 1.59, CI 1.02-2.46) than those not caught in the dust cloud. They were also more likely to report a physician diagnosis of a psychological condition (PR 2.59, CI 1.44-4.64). |
|  | McFarlane, 1987 | Volunteer fire-fighters | Exposure was correlated with GHQ score (p=0.007). ‘Cases’ on the GHQ differed significantly from non-cases in terms of exposure (p=0.02). However in regression analysis, exposure was not a significantly loading variable. |
|  | McFarlane, 1988b | Volunteer fire-fighters | The disordered (PTSD) and not-disordered groups were no different in their experience of the disaster as measured by exposure or perceived threat. |
|  | McFarlane & Papay, 1992 | Volunteer fire-fighters | Exposure did not appear to affect mental health outcomes.  Higher levels of exposure were experienced by both PTSD groups although no group differences emerged with Scheffe’s procedure (F(2127)=3.0, p<.05). |
|  | Misra et al., 2009 | London Ambulance Service personnel | Exposed personnel were more than twice as likely to have been affected (moderately or more) on a day-to-day basis (13% vs. 5%, p<0.05) and twice as likely to talk to others about the events (31% vs. 16%, p<0.01).  Those involved with response to the bombings were more likely to report probable PTSD (6% vs. 1%, p<0.05) and substantial stress (15% vs. 9%, not significant).  Amongst those involved, those with probable PTSD were more likely to have had a role on the disaster scene (42% vs. 15%, χ^2^=5.70, p<0.05).  Severity of casualties dealt with was not associated with outcomes. |
|  | North et al., 2002a | Fire-fighters | Exposure to children's remains and loss of family/friends were not associated with functional ability. |
|  | North et al., 2011 | WTC employees (above strike zone; mid-level floors; or lower floors); employees from organisations within 2 blocks of WTC; airline company employees; utility company employees involved in Ground Zero response; disaster response agency workers | Among those individuals not located within 0.1 mile of the towers, meeting PTSD symptom criteria was not associated with distance from the towers (Mann-Whitney z=−1.55, P=.123). Thus, geographical distance did not predict PTSD symptom criteria.  Among the group directly exposed to danger during the attacks, report of additional eye-witnessed experiences during the attacks was associated with higher rates of PTSD symptom criteria (47%, 31/66 vs 14%, 5/36 of others without eye-witnessed exposures; X^2^=11.16, df 1,P<.001). Additional exposure through a close associate’s direct exposure, however, did not increase the likelihood of meeting PTSD criteria among those physically endangered (X^2^=0.16, df 1, P=.692). |
|  | Perrin et al., 2007 | Recovery workers | Evacuating from one of the towers was associated with increased risk of PTSD in all professions except fire-fighters (OR 2.5 (p<0.01) for police, OR 2.2 (p<0.05) for emergency services personnel, OR 2.0 (p<0.05) for construction/engineering workers). |
|  | Pietrzak et al., 2012 | Police | Associated with full // subsyndromal PTSD respectively:  Total number of exposures (OR 1.16, 95% CI 1.25-1.48 // OR 1.22, 95% CI 1.16-1.28)  Caught in dust cloud (OR 1.43, 95% CI 1.07-1.91 // OR 1.54, 95% CI 1.29-1.85)  Involvement in search/rescue during 9/01-10/01 (1.37, 95% CI 1.01-1.85 // 1.35, 95% CI 1.11-1.63)  Not associated with full // subsyndromal PTSD respectively:  Working primarily or adjacent to pit during 9/01 (0.97, 95% CI 0.67-1.41 // 1.08, 95% CI 0.85-1.38)  Associated with full // subsyndromal PTSD respectively:  Exposure to human remains (OR 2.20, 95% CI 1.62-2.99 // OR 1.33, 95% CI 1.11-1.59) |
|  | Pietrzak et al., 2014 | WTC responders | Police: those with a severe chronic, delayed-onset or recovering trajectory were more likely than those with a resistant trajectory to report greater severity of WTC exposure: RRR (95% CI): 1.54 (1.40-1.68) severe chronic; 1.35 (1.26-1.44) delayed-onset; 1.29 (1.20-1.37) recovering.  Non-traditional responders: Those with severe chronic, delayed-onset, sub-syndromal increasing, moderate chronic & recovering trajectory were more likely than those in the resistant trajectory to report greater severity of WTC exposure RRR (95% CI): 1.25 (1.20-1.31) severe chronic; 1.10 (1.04-1.16) delayed onset; 1.09 (1.05-1.14) sub-syndromal increasing; 1.20 (1.14-1.26) moderate chronic; 1.19 (1.12-1.26) recovering.  Police & non-traditional responders: Exposure to human remains was associated with a severe chronic trajectory (statistics not shown).  Non-traditional responders: having worked adjacent to the pit/pile was associated with a severe chronic trajectory of PTSD (stats not reported). |
|  | Schwarzer et al., 2014 | Police officers | The simple mediation model to predict Wave 2 stress response from exposure levels, with Wave 1 stress as a mediator and sex and age as covariates, resulted in a significant mediation effect, Sobel z=9.84, p<.001. In addition to the main effects of social integration on stress responses, there was an interaction between exposure and social integration (p<.001), as well as an interaction between earlier stress responses and social integration on later stress responses (p<.001). Of the Wave 1 stress response variance, 12% were jointly predicted by exposure levels, social integration, and their interaction. Of the Wave 2 stress response variance, 48% were jointly predicted by the baseline, social integration, and their interaction. |
|  | Slottje et al., 2007 | Fire-fighters, police officers | Significant at the p<.05 level:  prevalence (%) of lower HRQoL, exposed v non-exposed respectively:  Physical symptoms total (33.2 v 10.3), physical limitation (53.9 v 22.2), bodily pain (35.6 v 13.9), unfavourable evaluation of health in general (12.3 v 1.0), role-physical (23.4 v 8.8), vitality (29.3 v 18.0).  Non-significant: Mental symptoms total (27.8 v 19.6), emotional limitation (6.3 v 3.1), social function (41.9 v 25.3), role-emotional (13.2 v 6.2) and role limitation (26.0 v 12.4). |
|  | Slottje et al., 2008 | Fire-fighters, police officers | The prevalence of PTSS was significantly higher among exposed compared with non-exposed police officers (6.5% v 2.4%, p<0.05).  Exposed workers reported multiple physical symptoms significantly more often than non-exposed colleagues; adding PTSS to the regression models did not change the effect of exposure status. No significant interactions between exposure status and PTSS were found (p-values of interactions ranged from .19 to .98). |
|  | Stewart et al., 2004 | Volunteer responders | Those who were human-remains-exposed were more likely to meet criteria for PTSD (71% v 17%; X^2^ (1)=3.90, p<.05).  There were no significant associations of human remains exposure with any of the criterion measures of drinking behaviour. |
|  | Thoresen et al., 2009 | NGOs, police, journalists, health personnel, ministry of foreign affairs, travel agencies, chaplains, air force medics | Level of intrusive memories was higher in disaster-area personnel than home-base personnel (5.4 vs. 4.2, p=0.02). Stress reactions were significantly associated with **witnessing experiences** (disaster-area group, unadjusted B value=1.36, adjusted B value=0.88, p<0.01 for both) and having to reject victims in need of help (both groups; disaster-area group unadjusted B value=2.37, adjusted B value=1.61, p<0.01 for both; stats for home-base group not shown). For disaster-area personnel all exposure variables were univariately associated with stress reactions. |
|  | Thormar et al., 2013 | Red Cross volunteers | Higher levels of exposure were predictive of anxiety (exposure scale consisted of loss of resources [β=.19, p<0.01], concern for others [β=.17, p<0.05], grotesque exposure [β=.25, p<0.01] and hours working [β=.17, p<0.05]).  Being exposed to grotesqueness was related to anxiety at 18-month follow-up (B=.33, β=.25, p<0.01). |
|  | Tucker et al., 2002 | Body handlers | Physical blast exposure not significantly correlated with posttraumatic stress. |
|  | Valenti et al., 2014 | Therapists who deliver direct care to persons with autism | Mean emotional exhaustion scores (exposed/non-exposed respectively):  Baseline: 9.7 / 9.7  1 year: 22.0 / 9.6  2 years: 15.5 / 9.3  Staff in the exposed group appeared to report significantly higher levels of emotional exhaustion after 1/2 years of follow-up. The exposure-by-time effect is significant (0.31; 66. 13; p<.0001).  Mean personal accomplishment scores (exposed/non-exposed respectively):  Baseline: 41.0 / 40.6  1 year: 33.4 / 40.8  2 years: 35.2 / 40.1  The exposed group show lower scores over time whereas the unexposed group remain stable. Exposure by time effect is significant, 0.24; 97.09; p<.0001.  Mean depersonalisation scores (exposed/non-exposed respectively):  Baseline: 2.8 / 2.3  1 year: 3.0 / 2.6  2 years: 3.2 / 3.0  Data show no significant differences between groups although there is a significant time effect for both groups. |
|  | Warren et al., 2003 | Emergency medicine physicians | NYC participants (exposed) reported more emotional distress than Wisconsin participants (non-exposed) although not significantly (t(106)=-1.87, p=0.7).  Positive effects: Practitioners in NY were more likely to report the attack had increased their awareness of psychological issues related to trauma (t(105)=-2.26, p<.05) and that the attack had increased their interest in learning more about psychological issues related to trauma (t(106)=-2.05, p<.05). |
|  | Weiss et al., 1995 | Emergency services personnel | Exposure during the critical incident was positively related to symptoms, but the relationship was weakest with the measure of general symptoms and stronger with the more specific measures of traumatic symptoms (r = .21 for intrusion, .19 for avoidance, .20 for hyperarousal, .20 for PTSD, .15 for general symptoms on SCL). |
|  | West et al., 2008 | Police | Recovery of bodies was a risk factor associated with PTSD (prevalence ratio 1.7, 95% CI 1.2, 2.3). |
|  | Witteveen et al., 2007 | Fire-fighters, police officers | Somatic complaints and fatigue symptoms were reported significantly more often (p<0.005) by exposed than non-exposed fire-fighters, after adjustment for potential confounders.  Exposed police officers reported significantly more symptoms of anxiety, depression, somatic complaints, sleep disturbance, fatigue and PTSD than non-exposed police (p<0.005). |
|  | Zhen et al., 2012 | Red Cross nurses | Nurses in the exposed group had significantly higher psychological complaints than controls: The exposed group reported a higher frequency of traumatic thought avoidance (70.0%, n = 147 vs.36.4%, n = 86), intrusive thoughts (57.1%, n = 120 vs. 33.9%, n = 80), startle (47.1%, n = 9) vs. 33.5%,n = 79), loss of pleasure (37.1%, n = 78 vs. 19.9%,n = 47), flashbacks (35.2%, n = 74 vs. 13.6%, n = 32), irritability (34.8%, n = 73 vs. 18.2%, n = 43), emotional numbing (29.5%, n = 62 vs. 19.1%, n = 45), and nightmares (26.7% n = 58 vs. 7.6%, n = 18; p = .000) than did non-exposed nurses. |
| DURING DISASTER: Duration on site and arrival time | Berninger et al., 2010a  Berninger et al., 2010b | Fire-fighters | *Duration on site:* Prolonged work of >4 months at World Trade Centre site significantly increased elevated PTSD risk (OR 2.0; 95% CI 1.8-2.3).  *Arrival time:* Earliest arrival at the World Trade Centre site significantly increased elevated PTSD risk (OR 6.0; 95% CI 4.4-8.3).  *Duration on site:* Prolonged work of >4 months at World Trade Centre site significantly increased elevated PTSD risk, OR 2.0 (CI 1.7-2.3).  *Arrival time:* Earliest arrival at the World Trade Centre site significantly increased elevated PTSD risk OR 4.8 (CI 3.0-7.5) |
|  | Bowler et al., 2012 | Police | *Duration on site:* Number of days worked at WTC site was not significantly associated with PCL scores. |
|  | Brackbill et al., 2009 | Rescue & office workers | *Duration on site:* Rescue/recovery workers who worked longer periods (>90 days) at the disaster site were at increased risk for PTS symptoms; AOR 1.6 [95% CI, 1.4-1.8].  *Arrival time:* Workers who began work at the start of disaster were at greater risk of PTS symptoms than those who arrived a week later, AOR 1.3 [95% CI, 1.1-1.5]. |
|  | Chiu et al., 2011 | Fire-fighters | *Arrival time:* Those who arrived morning of attack were 3.5 (CI 2.2-5.4) times more likely to develop depression and 4.9 (CI 3.0-7.9) times more likely to develop PTSD than arriving later. Significantly higher than even the afternoon of attack (depression = 1.9; [1.2-2.8] & 1.5; [1.0-2.4]; PTSD 2.4; [1.5-3.7] & 2.1; [1.3-3.3] in bivariate and multivariate analyses respectively). After adjusting for elevated PTSD risk, the magnitude of the associations for depression with exposure groups was substantially reduced (OR range: 1.2–1.6) and was no longer statistically significant. After adjusting for elevated depression risk as the new potential mediator, the magnitude of association between elevated PTSD risk and arriving on day 1 as opposed to day 2 onwards, remained similar, and statistically significant (p<0.01). |
|  | Dobashi et al., 2014 | Japan Ground Self-Defense Force Personnel | *Duration on site:* No significant differences found based on time spent working at the site. |
|  | Hagh-Shenas et al., 2005 | Rescue workers – fire-fighters, red crescent, student volunteers | *Duration on site:* Length of time on site was not associated with anxiety.  *Arrival time:* Arrival time at earthquake was not associated with anxiety. |
|  | Liao et al., 2002 | Rescue workers | *Arrival time:* Time of arrival at the scene had no significant predictive effect on psychological distress. |
|  | Luft et al., 2012 | Police & non-traditional responders | *Duration on site:* If long hours worked with respiratory problems, for police, 1.6 (1.5-1.8) times more likely to develop PTSD and for non-traditional responders, 2.0 (95% CI 1.8–2.2) more likely. |
|  | McFarlane, 1987 | Volunteer fire-fighters | *Duration on site:* Cases and non-cases on the GHQ differed significantly in terms of hours spent fire fighting (p=0.003). |
|  | Misra et al., 2009 | London Ambulance Service personnel | *Arrival time:* Time of arrival on the scene was not associated with outcomes. |
|  | North et al., 2002a | Fire-fighters | *Duration on site:* Change in job satisfaction for the worse was associated with greater number of days worked at the site (Wilcoxon X^2^=6.34, p<.05). |
|  | North et al., 2002b | Fire-fighters | *Duration on site:* PTSD was associated with more time spent at the bombing site (mean=10.0 days vs 7.9 days for those without PTSD, t=2.41, df=180, p<0.02).  PTSD was associated with more time spent in the pit (mean=21.7 hours v 9.6 hours for those without PTSD, t=2.64, df=89, p=0.01). |
|  | Perrin et al., 2007 | Various rescue and recovery workers: 3925 police; 3232 fire-fighters; 1741 emergency medical services; 4498 construction/engineering; 1798 sanitation workers; 5438 volunteer organisations e.g. Red Cross; 3797 unaffiliated volunteers e.g clergy and finance workers; 4263 other government agencies | *Arrival time:* Earlier start date and longer duration of time worked at the WTC site were significant risk factors for PTSD for all occupations except police.  Fire-fighters: those who started September 11th (p<0.001) or 12th (p<0.05) more likely to have PTSD  Emergency personnel: those who started September 11th (p<0.0001) more likely to have PTSD  Construction/engineering personnel: those who started September 11th (p<0.0001) more likely to have PTSD  Sanitation workers: those who started September 11th (p<0.01) or 12th (p<0.05) more likely to have PTSD |
|  | Pietrzak et al., 2012 | Police | *Duration on site:* Not associated with full // subsyndromal PTSD respectively:  Total hours worked greater than median (1.26, 95% CI 0.96-1.65 // 1.14 (95% CI 0.96-1.35).  *Arrival time:* Associated with full // subsyndromal PTSD respectively:  Arrived on 9/11 or 9/12 (OR 1.80, 95% CI 1.11-2.89 // OR 1.59, 95% CI 1.19-2.13) |
|  | Pietrzak et al., 2014 | WTC responders | *Duration on site:* Non-traditional responders: Working more than the median number of hours at the WTC site was associated with each of the PTSD symptom trajectories –  Relative risk ratio (95% CI):  Severe chronic: 2.05 (1.67-2.43), p<0.001  Delayed-onset: 1.83 (1.46-2.19), p<0.001  Subsyndromal increasing: 1.30 (1.09-1.52), p<0.01  Moderate chronic: 1.73 (1.38-2.07), p<0.001  Recovering: 1.61 (1.25-1.98), p<0.001  *Arrival time:* Non-traditional responders: Early arrival was negatively associated with PTSD (relative risk ratio 0.50 (95% CI 0.40-0.60), p<0.001 for severe chronic PTSD; RRR 0.68 (95% CI 0.53-0.83, p<0.001 for delayed-onset, RRR 0.60 (95% CI 0.47-0.73), p<0.001 for moderate chronic, RRR 0.73 (95% CI 0.54-0.91), p<0.05 for recovering) |
|  | Saroja et al., 1995 | Fire-fighters | *Duration on site:* The authors compared total direct exposure to the disaster in hours between subjects with high IES scores (32.5%) and subjects with low IES scores (67.5%). Total direct exposure in hours was 66 for those with high scores and 70 for those with low scores; this difference was not significant. |
|  | Soo et al., 2011 | Fire-fighters | *Arrival time:* Prevalence of probable PTSD, by arrival group:  2006-2007: 14% arrival group 1 (those who arrived during the morning of 9/11), 6.7% group 2 (who arrived during the afternoon of 9/11), 6.5% group 3 (who arrived on day 2), 5.0% group 4 (who arrived at any time between days 3-14).  2007-2008: 10.9% group 1, 5.7% group 2, 4.9% group 3, 5.4% group 4.  2008-2009: 12.8% group 1, 6.5% group 2, 5.2% group 3, 4.7% group 4.  2009-2010: 13.4% group 1, 6.5% group 2, 6.5% group 3, 5.4% group 4.  The prevalence of PTSD was significantly associated with arrival group in all 4 years of analyses (p<.0001). |
|  | Stellman et al., 2008 | WTC workers | *Duration on site:* % of PTSD, depression & panic cases respectively, by days at site:  Up to 2 weeks: 9.1%, 8.2%, 4.9%  Up to 1.5 months: 9.1%, 6.6%, 4.7%  Up to 3 months: 11.5%, 9.4%, 5.3%  Up to 5.5 months: 11.9%, 9.4%, 4.9%  More than 5.5 months: 13.3%, 10.6%, 5.3%  Days at site was predictive of PTSD and depression (p<.001) but not panic.  % of PTSD, depression & panic cases respectively, for those present/not present on 9/11-9/12:  Present: 10.2%, 6.3%, 4.5%  Not present: 12.5%, 12.8%, 5.8% (all significant p<.001). |
|  | Thormar et al., 2013 | Red Cross volunteers | *Duration on site:* Working long hours on site related to anxiety (B=.14, β=.17, p<0.01) and subjective health symptoms (B=1.28, β=.19, p<0.05). |
|  | Yokoyama et al., 2014 | Dispatched health nurses | *Duration on site:* The factor that most strongly influenced subjective well-being, low mood, worsened sleep state, and intense fatigue was work hours per day at the dispatch destination. Logistic regression analysis showed that the health of dispatched health nurses who worked more than 8 h per day was 1.76–2.11 times more likely to be adversely affected than that of the nurses who worked less than 8 h. |
| DURING DISASTER: Emotional involvement | Cetin et al., 2005 | Military rescue workers | In the rescue group, identification with the deceased with self (X^2^=32.74, p<0.01), friend (X^2^=11.01, p<0.01) and family (X^2^=31.11, p<0.01) was sig higher than in control group. Identification with deceased with self, friends and family, were all significant negatively correlated with intrusion, avoidance, arousal and IES total (p<0.01). Identification–Friend occurred less often (47.2%) than Identification–Self (85.7%) and Identification–Family (85.4%) did, but was more strongly associated with PTSD symptom scores (-0.201; -0.186; -.0186, p<0.01 respectively). |
|  | Eidelson et al., 2003 | Psychologists | Regression analyses = Increased stress was significantly positively associated with client distress (β =0.16, p<0.001) |
|  | Hodgkinson & Shepherd, 1994 | Social Workers | Only one symptom measure distinguished the high and low identification groups, this being the HSCL Obsessive/ Compulsive subscale (t = 2.25, p<0.05), indicating that the high identification group had a tendency to greater cognitive disturbance such as unpleasant intrusive thoughts. |
|  | Ursano et al., 1999 | Volunteer body handlers | Of the subjects who identified with the deceased as a friend, 33% (N=3) had PTSD at some time during the follow-up, compared with only 4.5% (N=1) of the Non-identifiers (χ2=4.71, df=1, p=0.03). Identification with deceased as family member associated with greater intrusive symptoms: One month after the disaster, the scores on intrusion were higher for those who reported identifying with the deceased as a family member than those who did not (mean 12.9 vs. 7.6). |
| DURING DISASTER: Peri-traumatic distress/dissociation | Ben-Ezra et al., 2006a | Rescue Personnel | For men and women analysed separately and together, no significant correlations found between peri-traumatic dissociation, and intrusion, avoidance or PTS symptoms. |
|  | Ben-Ezra et al., 2006b | Rescue Personnel | Increased peri-traumatic dissociation was the only significant change when comparing rescue personnel before and after disaster (Wilcoxon z =−2.023, p<0.05). The failure to find any post-traumatic reactions may suggest that the first impact of a traumatic event leads to increased dissociation later followed by post-traumatic reactions. Mapping the level of dissociation at an early stage may show if there is a direct link to subsequent PTSD. |
|  | Biggs et al., 2010 | Range of rescue personnel | The risk of probable ASD increased (OR = 1.88) with increasing numbers of reported peri-traumatic dissociative symptoms (Wald X^2^= 15.84, p<0.001, CI = 1.38–2.56). |
|  | Cardozo et al., 2012 | Humanitarian Aid Workers | Extraordinary peri-traumatic stress contributed to increased burnout (AOR 1.5; CI 1.17-1.83), chronic stress during deployment contributed to increased risk for depression post deployment (AOR 1.1; CI1.02-1.20). |
|  | Fullerton et al., 2004 | Rescue workers | Early dissociative symptoms significantly increased likelihood of PTSD. Number of dissociative symptoms increased likelihood by 1.82 (CI 1.25-2.66). |
|  | Grieger et al., 2003 | Military & civilian hospital staff | Respondents with acute stress disorder had higher levels of peri-traumatic dissociation (U=555, Z=–7.964, p< .001). Increased alcohol use reported higher levels of peri-traumatic dissociation (U=1514, Z=–2.39, p=.017). |
|  | Gross et al., 2006 | Clean up & Recovery workers | Exposed workers with PTSD were 4.47 times more likely to report peri-event anxiety (CI 3.17-6.30, p<0.0001). |
|  | McFarlane, 1988a | Volunteer fire-fighters | Regression analysis of the IES score against GHQ score demonstrated that distress generated by the disaster accounted for 14% of the variance of psychiatric impairment (F=55.6, df=1333, β =0.32, p<0.000). |
|  | Morren et al., 2005 | Volunteer fire-fighters | Having experienced a personal distressing event was associated with more hyperarousal (β =0.16; CI, 0.04-0.28), re-experiencing (β =0.20, CI, 0.07-0.33) and total PTSD score (β =0.21, CI, 0.09-0.33). Positive relationships emerged between direct tasks and re-experiencing (β =0.16, CI, 0.00-0.31) and between disaster-related events and avoidance (β =0.22; CI, 0.07–0.37), hyperarousal (β =0.16; CI, 0.01–0.30), re-experiencing (β =0.21; CI, 0.05–0.37), and total post-traumatic stress (β =0.24; CI, 0.09–0.38).  While none of the disaster-related exposure variables significantly predicted SCL scores, personal distressing events predicted anxiety (β =0.23; CI, 0.11-0.34), depression and hostility (both β =0.22; CI, 0.10-0.33) and sleeplessness (β =0.18; CI, 0.06–0.29). Work-related events predicted anxiety (β =0.13; CI, 0.00–0.26), depression (β =0.18; CI, 0.06–0.31), and sleeplessness (β =0.14; CI, 0.01–0.27). Only marginal significance was attained for the positive association between depression, hostility, and sleeplessness, on the one hand, and disaster-related events, on the other (βs range from 0.13 to 0.14; p<0.10). |
|  | Weiss et al., 1995 | Emergency services personnel | For all five indices of symptomatic distress, general dissociative tendencies measured using the DES and peri-traumatic dissociation at the time of the critical incident as measured by the PDEQ-SR accounted for significant increments in symptom variance beyond the first set of 5 predictors, which themselves accounted for a significant amount of variability as a set.  Both measures of dissociation were strongly positively correlated with general symptoms but were also substantially related to intrusion, avoidance, hyperarousal, and overall PTSD symptoms as well. |
| DURING DISASTER: Role-related stressors | Adams et al., 2008 | Social Workers | Bivariate associations - Those who reported having enough information to perform their work effectively had lower secondary trauma (B=-0.14, SE=0.05, β = –0.19, p<.01). The higher respondents scored on the mastery measure, the lower their job burnout scores (B=-0.21, SE=0.05, β = –.26, p<.001). For the work information variable, having such information tended to lower job burnout but was non-significant (B= –0.10, SE=0.06, β =0.12, p<.10) |
|  | Berninger et al., 2010a  Berninger et al., 2010b | Fire-fighters | Providing supervision without previous supervisory experience significantly increased elevated PTSD risk (OR 4.1; 95% CI 2.8-6.1).  As above, OR 2.2 (CI 1.7-2.9)  Significantly greater proportion of elevated PTSD risk in fire-fighters who normally did not have supervisory responsibilities but did during the disaster compared to line managers who had already had training and experience (OR 2.4; 95% CI 1.5-3.7). |
|  | Creamer & Liddle, 2005 | Disaster mental health workers | Secondary traumatic stress was associated with longer assignments (r=0.27, p<0.05), increased time spent with child clients (r=0.28, p<0.05) & clients who discussed morbid material (r=0.29 & r=0.31, p<0.05). Those who worked with fire-fighters had significantly more STS (t(78)=2.23, p<0.05). |
|  | Ehring et al., 2011 | Recovery workers | Symptom levels of PTSD were associated with work-related stressors (r = 0.27, p<0.001). |
|  | Fullerton et al., 2013 | Public health workers | Those with high work demand had increased alcohol (OR=1.98) & tobacco use (OR=2.1). The regression model, which included exposure, work demand, and the demographic variables, those with high work demand continued to be at increased risk of increased alcohol use for at least a 2-week period (OR=1.70; X^2^=4.01, df=1, P<.045, 95% CI=1.01-2.85). Work demand was not associated with probable PTSD or depression. |
|  | Hodgkinson & Shepherd, 1994 | Social Workers | High reporting of role problems (ambiguity, conflict, overload) was associated with poorer outcomes in wellbeing (somatisation - t=3.22, p<0.01; Obsessive/compulsive - t=3.65, p=0.001; depression - t=2.45, p<0.05; preoccupation - t=2.21, p<0.05).  There were no significant results in relation to high caseload and psychological distress. |
|  | Murphy et al., 2004 | Fire-fighters | Five job threat variables were significantly correlated with IES score 6months pre-disaster: **not being able to predict/control events (p<0.01), lack of control over nature/extent of victim injuries (p<0.001),** worries about personal competence in handling mass casualty runs (p<0.05), exposure to increased personal risk (p<0.001) and concerns about personal injury/disability/death due to work (p<0.05). One week after 9/11 the same concerns were correlated but the magnitude of the relationships were slightly stronger especially for exposure to personal risk and concerns about personal injury/death. Associations were found with IES score and **not being able to predict/control events (p<0.001),** worries regarding team competence when responding to disaster (p<0.001), **lack of control over victim injuries (p<0.001),** worries regarding personal competence in handling mass casualty runs (p<0.01), exposure to increased personal risk (p<0.001), concerns about personal injury/death/disability (p<0.001). |
|  | Perrin et al., 2007 | Various rescue and recovery workers: 3925 police; 3232 fire-fighters; 1741 emergency medical services; 4498 construction/engineering; 1798 sanitation workers; 5438 volunteer organisations e.g. Red Cross; 3797 unaffiliated volunteers e.g clergy and finance workers; 4263 other government agencies | Prevalence of PTSD highest among those who performed tasks not common for their occupation. E.g.: Fire fighting was associated with increased risk of PTSD in police (OR 2.1 (95% CI 1.0-4.2), p<0.05) and emergency services (OR 2.4 (95% CI 1.5-4.1), p<0.001); performing light construction was the only task associated with PTSD in fire-fighters (OR 1.4 (95% CI 1.1-1.9), p<0.05). |
|  | Sloan et al., 1994 | Police, fire, medical and mental health personnel involved in emergency service work | Self-reported qualitatively heavy workload predicted intrusiveness and avoidance of thoughts both immediately after and at the 6-month anniversary of the incident. Regression analyses showed that qualitative workload was the only significant predictor of intrusion scores at T1 and T2, and avoidance scores at T2 (R=.55, .66 and .42, p<.001, respectively). Additionally, time pressure and quantitatively heavy work load also predicted avoidance score at the time of the incident: time pressure was most predictive followed by quantitative and then qualitative workload (overall R=.45, p<.001). |
|  | Thoresen et al., 2009 | NGOs, police, journalists, health personnel, ministry of foreign affairs, travel agencies, chaplains, air force medics | Stress reactions were significantly associated with witnessing experiences (disaster-area group) and **having to reject victims in need of help** (both groups) (adjusted B value 1.61, p<0.01 for disaster-area group; stats for home-base group not shown). |
|  | Thormar et al., 2013 | Red Cross volunteers | Anxiety increased in relation to lack of information received about the situation (B=-64, β=-.25, p<0.001).  PTSD: providing PSS (psychosocial support) and food aid to affected community were significant contributors to symptoms (B=-13.93, β=-.25, p<0.001 for providing PSS; B=-6.07, β=-.19, p<0.01 for providing food aid). Specific tasks performed did not predict anxiety. Providing PSS, food aid and handling administration predicted depression (B=-1.85, β=-.15, p<0.05 for providing PSS, B=-1.14, β=-.17, p<0.05 for providing food aid, B=-1.08, β=-.16, p<0.05 for administration). |
|  | Warren et al., 2003 | Emergency medicine practitioners | Emotional distress was significantly associated with whether they treated an individual injured in the attack (t(105)=2.46, p<.05). |
|  | West et al., 2008 | Police | Being involved with crowd control was associated with PTSD (prevalence ratio 1.6, 95% CI 1.1, 2.1). |
|  | Witteveen et al., 2007 | Fire-fighters, police | In regression analysis tasks performed at disaster site were associated with SRIP total (B=1.03).  Relevant associations in exposed police included: A1 events, A1 tasks and other tasks with disaster-related posttraumatic distress on IES total (B=1.38; B=1.31; B=1.13). |
| DURING DISASTER: Perceptions of safety, threat and risk | Cukor et al., 2011 | Non-rescue / utility workers | Single best predictor of PTSD was the subjective perception of danger to oneself, not actual occupational or personal exposure.  Risk of probable PTSD was 1.83 (p<0.001) times higher in those with trauma history, and 2.46 (p<0.001) for those with trauma history and who felt their life was in danger. |
|  | Grieger et al., 2003 | Military & civilian hospital staff | Respondents with acute stress disorder endorsed lower perceptions of current safety (at time of assessment after disaster; U=2,045, Z=–4.348, p<.001) and higher levels of depressive symptoms (U=884, Z=–6.167, p< .001) compared with those without acute stress disorder. |
|  | Jayasinghe et al., 2008 (FU of Evans et al., 2006) | Disaster workers | Multiple regression analyses found that feeling in serious danger was sig. predictor of PTSD severity at time 2 (1 year FU), β = 0.22, t=6.93, p<0.001. |
|  | Marmar et al., 1996 | Emergency personnel | Participants above the threshold for ‘caseness’ on the SCL-90-R reported greater feelings of threat (p<.01). |
|  | Matsuoka et al., 2012 | Disaster Medical Assistance Team workers | Concern over radiation exposure was strongly associated with distress. Both men and women with radiation concerns showed significantly higher K6, CES-D, PDI, and IES-R scores than in those without concern. After controlling for age, occupation, disaster operation experience, duration of time spent watching earthquake news, and past history of psychiatric illness, these associations remained significant in men, but did not remain significant in women for the CES-D and PDI scores. In addition, after excluding those with past history of psychiatric illness, ANOVA results showed no apparent change in the association between concern over radiation and psychological distress in men. In women, however, the association remained significant for the K6 (p=.029) but not for the CES-D, PDI, or IES-R (p=.097, p=.064, p=.064). |
|  | McFarlane, 1987 | Volunteer fire-fighters | Perceived threat was significantly correlated with GHQ score (p=0.004) and caseness on the GHQ (p=0.01) but was not a significantly loading variable in regression analysis. |
|  | McFarlane, 1988b | Volunteer fire-fighters | The disordered (PTSD) and not-disordered groups were no different in their experience of the disaster as measured by perceived threat. |
|  | Murphy et al., 2004 | Fire-fighters | Five job threat variables were significantly correlated with IES score 6months pre-disaster: not being able to predict/control events (p<0.01), lack of control over nature/extent of victim injuries (p<0.001), worries about personal competence in handling mass casualty runs (p<0.05), **exposure to increased personal risk (p<0.001) and concerns about personal injury/disability/death due to work (p<0.05).** One week after 9/11 the same concerns were correlated but the magnitude of the relationships were slightly stronger especially for **exposure to personal risk and concerns about personal injury/death.** Associations were found with IES score and not being able to predict/control events (p<0.001), worries regarding team competence when responding to disaster (p<0.001), lack of control over victim injuries (p<0.001), worries regarding personal competence in handling mass casualty runs (p<0.01), **exposure to increased personal risk (p<0.001), concerns about personal injury/death/disability (p<0.001).** |
|  | Thormar et al., 2013 | Red Cross volunteers | Being concerned for others in immediate aftermath related to anxiety at 18months (B=.20, β=.17, p<0.05). Worries about personal safety were predictive of PTSD (B=4.44, β=.22, p<0.01) and anxiety (B=.71, β=.26, p<0.001). Concern about the quality of equipment also predicted anxiety (B=-.46, β=-.20, p<0.05). |
|  | Wang et al., 2010 | Healthcare workers | The significant risk factors associated with PTSD severity included being female (B = 3.85, β = .11, t = 2.07, p < .05), being injured (B = 5.67, β = .12, t = 2.36, p < .05), being bereaved (B = 5.66, β = .18, t = 3.49, p < .01), and **intensity of initial fear** (B = 3.01, β = .22, t = 4.19, p < .01). |
| DURING DISASTER: Harm to self or close others | Berninger et al., 2010a | Fire-fighters | For each additional death of a colleague, there was a corresponding 10.0% increase in the odds of ever having elevated PTSD risk (OR 1.1; 95% CI 1.1-1.2). |
|  | Corrigan et al., 2009 | Fire-fighters | Loss of a co-worker led to a near 4 fold increase in elevated PTSD (OR=3.71, CI 2.93-4.70, p<0.001) & more than a 2 fold increase in counselling (OR=2.56, CI 2.24-2.93, p<0.001) – after adjusting for years as a fire fighter. |
|  | Huang et al., 2013 | Military rescue workers | Subjects who themselves survived death, were seriously injured or had severe mental trauma had a rate of PTSD 25.6 times higher than those who did not (OR = 25.6, 95%CI: 13.9–47.3).  Those who experienced death of family members suffered from PTSD symptoms at a rate 4.7 times greater than those who did not (OR: 4.71, 95%CI: 1.83–12.15). |
|  | Luce et al., 2002 | Health Service workers | Having someone close killed/injured was associated with high PTSD score when other involvement was also present, but associated with lowest PTSD when grief alone – no statistics presented. |
|  | Luft et al., 2012 | Police & non-traditional responders | Abnormal lung function as a result of dust from disaster was not significantly associated with probable PTSD. Respiratory symptoms were substantially associated with probable PTSD (r=0.28 in police and 0.27 in non-traditional responders). |
|  | McFarlane, 1987 | Volunteer fire-fighters | Injury was not significantly correlated with GHQ score (p=0.3) or GHQ caseness (p=0.5) nor significant in regression analysis. |
|  | McFarlane, 1988b | Volunteer fire-fighters | The disordered (PTSD) and not-disordered groups were no different in their experience of the disaster as measured by injury. |
|  | McFarlane & Papay, 1992 | Volunteer fire-fighters | Injury did not appear to affect mental health outcomes. |
|  | North et al., 2002b | Fire-fighters | PTSD was not associated with injury. |
|  | Perrin et al., 2007 | Recovery workers | Sustaining an injury was the only within-disaster experience which increased risk of PTSD and was strongest risk factor for all except police and construction (Police: OR 2.4, 95% CI 1.3-4.7, p<0.0001; Fire-fighters: OR 2.7, 95% CI 1.9-3.9, p<0.0001; Emergency service personnel: OR 4.0, 95% CI 2.8-5.7, p<0.0001; Construction/engineering workers: OR 1.9, 95% CI 1.6-2.3, p<0.0001; Sanitation workers: OR 2.0, 95% CI 1.4-2.8, p<0.0001). |
|  | Pietrzak et al., 2012 | Police | Associated with full // subsyndromal PTSD respectively:  Losing someone on 9/11 (2.10, 95% CI 1.75-2.52 // 2.10, 95% CI 1.75-2.52) |
|  | Pietrzak et al., 2014 | WTC responders | Police: Enduring an illness or sustaining an injury while working at the WTC site was associated with severe chronic, delayed-onset and recovering trajectories (RRR (95% CI) 1.63 (1.37-1.90), p<0.001; 1.43 (1.25-1.61), p<0.001; 1.31 (1.14-1.48), p<0.001 respectively).  Non-traditional responders: Enduring an illness or sustaining an injury at the site was associated with each of the symptom trajectories –  RRR (95% CI):  Severe chronic: 1.37 (1.24-1.49), p<0.001  Delayed onset: 1.21 (1.08-1.34), p<0.001  Subsyndromal increasing: 1.18 (1.07-1.29), p<0.001  Moderate chronic: 1.34 (1.20-1.48), p<0.001  Recovering: 1.22 (1.08-1.37), p<0.001  Police: Knowing someone injured was associated with severe chronic, delayed-onset and recovering trajectories. Traumatic loss of friend/family/colleague were additionally associated with a severe chronic trajectory.  Non-traditional responders: Knowing someone who was injured at the site/traumatic loss of colleague or friend or family member were associated with a severe chronic trajectory. (Stats not reported.) |
|  | Stellman et al., 2008 | WTC workers | Significantly elevated rates of PTSD [OR=1.66; 95% CI, 1.21-2.28] and emotional disability [OR=1.48, 95% CI, 1.16-1.87] associated with having lost a family member to the attack. Loss of friends resulted in a significant but smaller elevated risk for probable PTSD only [OR=1.2, 95% CI, 1.02-1.43]. |
|  | Tak et al., 2007 | Fire-fighters | In multivariate analysis adjusting for all covariates in the model, persons with new-onset lower respiratory symptoms had significantly higher risk of depressive symptoms (PR=1.8; 95% CI:1.2, 3.0) compared to those without new-onset respiratory symptoms. Depressive symptoms were also associated with skin rash (PR=1.7; 95% CI: 1.2, 2.6). |
|  | Tucker et al., 2002 | Body handlers | Knowing anyone killed or injured was not significantly correlated with posttraumatic stress. |
|  | Ursano et al., 2014 | Public health workers | Having high injury/damage was related to a significant increase in PTSD symptom severity.  Individual-level collective efficacy: Using a model that included all covariates, analyses revealed that an increase in individual-level collective efficacy was associated with a decreased probability of having probable PTSD (OR=0.94, CI=0.92–0.97). Further, **having high injury/damage increased the probability of being diagnosed with probable PTSD (OR=2.63, CI=1.33–5.21).**  Of those with high injury/damage, the probability of having PTSD is expected to be 0.12 if a person has a lower level of individual-level collective efficacy (score=20). This risk decreases sharply to 0.05 if he or she has a higher level individual-level collective efficacy (score=40), which is a 59% reduction. For those with lower injury/damage, the risk of having PTSD is expected to drop from 0.06 with a lower level of individual-level collective efficacy to 0.02 with a higher level of individual-level collective efficacy, which is a 67% reduction.  Community-level collective efficacy: In a model that contains all covariates, analyses revealed that an increase in community-level collective efficacy was associated with a decreased probability of having probable PTSD (OR=0.93, CI=0.88–0.98). Further, **having high injury/damage increased the probability of being diagnosed with probable PTSD (OR=2.29, CI=1.19–4.39).** |
|  | VanDevanter et al., 2014 | Nurses | Deployed nurses who also experienced personal storm-related loss, damage or **injury** were no more likely to report deployment-related stress than nurses who did not experience a personal storm-related event. |
|  | Wang et al., 2010 | Healthcare workers | The significant risk factors associated with PTSD severity included being female (B = 3.85, β = .11, t = 2.07, p < .05), **being injured** (B = 5.67, β = .12, t = 2.36, p < .05), **being bereaved** (B = 5.66, β = .18, t = 3.49, p < .01), and intensity of initial fear (B = 3.01, β = .22, t = 4.19, p < .01). |
|  | Warren et al., 2003 | Emergency medicine practitioners | Knowing someone injured in the attacks was associated with distress (t(106)=2.29, p<.05). |
|  | West et al., 2008 | Police | Injury to a family member was associated with PTSD and depression (PR 1.7, 95% CI 1.2, 2.4). |
| DURING DISASTER: Social support | Adams et al., 2008 | Social Workers | Multivariate associations - Those with higher social support and a higher sense of professional mastery were less distressed (B= –0.05, SE=0.02, β=-0.13, p<.05; B = –0.11, SE=0.03, β=-0.23, p<.001; respectively). |
|  | Bartone et al., 1989 | Military - Survivor Assistance Workers | Workers low in hardiness and social support report more illness than those high in these factors (p<0.05). |
|  | Biggs et al., 2014 | Police Officers | Exposure to certain aspects of a disaster was significantly associated with work culture support, which was in turn associated with job satisfaction, work engagement, psychological strain & turnover intentions (X^2^(1094)=2484.03, p<0.001).  Bivariate associations:  Work culture support significantly positively correlated at Time 1 and Time 2 respectively, with intrinsic satisfaction (0.61, 0.51); level of work engagement (0.45, 0.42); and supervisor support (0.35, 0.29), all p<0.001.  Work culture support significantly negatively correlated at T1 and T2 respectively, with turnover intentions (-0.41, -0.37); and psychological strain (-0.29, -0.21), all p<0.001, and frontline deployment (-0.08, p<0.01).  Supervisor support significantly positively correlated at T1 and T2 respectively, with intrinsic satisfaction (0.49, 0.36); level of work engagement (0.36, 0.28).  Supervisor support significantly negatively correlated at T1 and T2 respectively, with turnover intentions (-0.28, -0.23); and psychological strain (-0.28, -0.18), all p<0.001.  Psychological strain significantly positively correlated at T1 and T2 respectively, with job demands (0.15, 0.13); and turnover intentions (0.39, 0.31), all p<0.001.  Psychological strain significantly negatively correlated at T1 and T2 respectively, with job control (-0.27, -0.24); supervisor support (-0.28, -0.22); work culture support (-0.29, -0.26); intrinsic satisfaction (-0.42, -0.32); and level of work engagement (-0.39, -0.26).  Strongest predictors of T2 (1 month post) work-related characteristics and outcomes, were the baselines measures of each at T1 (10 months prior), demonstrating stability over time. |
|  | Boscarino et al., 2004 | Social Workers | Secondary traumatisation was negatively associated with supportive work environment (b=-0.151, SE=0.052, Beta= -0.196, p<0.01) as was job burnout (b=-0.136, SE=0.61, Beta=-0.180, p<0.01). |
|  | Brackbill et al., 2009 | Rescue & office workers | Social support was inversely related to PTSD risk. The greatest effect was observed among rescue/ recovery workers who reported no sources of social support compared with those who reported 4 or 5 sources (AOR, 6.9 [95% CI, 5.7-8.3]), similar for local office workers (AOR 5.3 [95% CI, 4.1-6.8). |
|  | Cardozo et al., 2012 | Humanitarian Aid Workers | Social support was associated with lower levels of depression (AOR 0.9; CI 0.84-0.95), psychological distress (AOR0.9; CI 0.85-0.97), burnout, lack of accomplishment (AOR 0.95; CI 0.91-0.98) & greater life satisfaction (p=0.0213). |
|  | Ehring et al., 2011 | Recovery workers | Symptom levels of PTSD were associated with low social support (r = -0.19, p<0.05).  High levels of social support were associated with less symptom severity on IES (r = -0.19, p<0.05), PADQ anx (r = -0.23, p<0.001), PADQ dep (r = -0.16, p<0.05), and MBI (r = -0.2, p<0.01). |
|  | Hodgkinson & Shepherd, 1994 | Social Workers | Low social support associated with poorer outcomes/greater symptomatology (obsessive/compulsive - t=2.06, p<0.05; preoccupation - t=2.01, p<0.05) - 58% mentioned sharing experiences with colleagues, very few mentioned work supervision (percentage unknown). |
|  | Holtz et al., 2002 | Human Rights Workers | Compared to excellent support, fair/poor organisation support was found to negatively affect mental health outcomes in depression (OR 1.1), PTSD (OR 1.2) and anxiety (OR 3.8), although this was not a significant effect. |
|  | Huang et al., 2013 | Military rescue workers | Correlations suggest PTSD was strongly positively correlated with social support (r = 0.58, p<0.01). Low social support had largest odd ratio in multivariate logistic regression, 20.42 (95% CI, 9.36-44.54, p<0.001). |
|  | Jenkins, 1996 | Emergency medical workers | Negative correlation between hostility & having social support available post disaster (r=-0.42, p<0.05). Positive correlation between Obsessive/Compulsive & hours spent with family post-disaster (r=0.48, p<0.01) and others understanding their experiences post-disaster (r=0.49, p<0.01) & at FU (r=0.39, p<0.05). |
|  | Jenkins, 1997a | Emergency workers | Social-emotional support was strongly positively correlated with treatment seeking (r=0.67, p<0.001), cognitive behavioural approach (r=0.62, p<0.001) & social approach (r=0.43, p<0.01). |
|  | Jenkins, 1997b | Fire dispatchers; 911 operators; police dispatchers | In multiple regression analyses, social support explained 10% of variance in intrusion. |
|  | Kaspersen et al., 2003 | UN soldiers and relief workers | For UN soldiers, social support was important for low trauma exposure, where as in relief workers, it was important for high exposure.  In UN soldiers, only colleague index had a significant effect on post-trauma symptoms (B = -1.57, SE B = 0.59, β = -1.95, p<0.01) and friends acted as a sig moderator on relation between trauma exposure & intrusion symptoms (B = 8.14, SE B = 2.95, β = 2.5, p<0.01).  In relief workers, no significant differences in moderator/buffer effects were found in their social network. UN soldiers were more trauma-exposed, but exhibited less PTSS symptoms. |
|  | Misra et al., 2009 | London Ambulance Service personnel | Level of awareness of support measures was high and did not differ between those with/without probable PTSD. |
|  | Murphy et al., 2004 | Fire-fighters | Satisfaction with work support and home support were not correlated with the IES measure. |
|  | North et al., 2002a | Fire-fighters | Fire-fighters with PTSD were less likely to report getting along with their boss (67% vs 85%, p<.05) and co-workers (54% vs 78%, X^2^=6.12, p<.05). |
|  | Pietrzak et al., 2012 | Police | Social support was associated with full PTSD but not subsyndromal PTSD:  Number of sources of work support while working at WTC site: OR 0.75, 95% CI 0.63-0.91 (significant) // 1.01, 95% CI 0.91-1.13 (not significant)  Number of sources of family support while working at WTC site: OR 0.80, 95% CI 0.73-0.86 (significant) // 0.99 (95% CI 0.94-1.04 (not significant) |
|  | Pietrzak et al., 2014 | WTC responders | Police: In the severe-chronic group, greater number of sources of family support while working at the WTC site was protective. In the delayed-onset group, having one or two sources of work support was protective.  Non-traditional responders: Greater family & work support were protective for the severe & moderate chronic groups. Greater family support was protective for the delayed-onset group. |
|  | Schwarzer et al., 2014 | Police officers | The simple mediation model to predict Wave 2 stress response from exposure levels, with Wave 1 stress as a mediator and sex and age as covariates, resulted in a significant mediation effect, Sobel z=9.84, p<.001. In addition to the main effects of **social integration** on stress responses, there was an interaction between exposure and social integration (p<.001), as well as an interaction between earlier stress responses and social integration on later stress responses (p<.001). Of the Wave 1 stress response variance, 12% were jointly predicted by exposure levels, social integration, and their interaction. Of the Wave 2 stress response variance, 48% were jointly predicted by the baseline, social integration, and their interaction. |
|  | Soffer et al., 2011 | Rescue workers | Social support was not correlated with either peri-traumatic dissociation or PTSD symptoms. |
|  | Tak et al., 2007 | Fire-fighters | Depressive symptoms were associated with dissatisfaction with supervisory support (PR=1.6; 95% CI: 1.1, 2.3).  Fire-fighters who answered that they were currently living with their families were less likely to report depressive symptoms (PR=0.7; 95% CI: 0.5, 1.0) than those not living with their families. |
|  | Thoresen et al., 2009 | NGOs, police, journalists, health personnel, ministry of foreign affairs, travel agencies, chaplains, air force medics | For home-base personnel, **being a target of harassment** (unadjusted B value 1.86, p<0.01; adjusted B value 0.46) and rejecting disaster victims in need of help were univariately associated with stress. |
|  | Thormar et al., 2013 | Red Cross volunteers | High need for support and lack of support from organisation in the aftermath were the strongest contributors of depression (B=.17, β=.17, p<0.05) and also predicted PTSD (B=.96, β=.21, p<0.01) and anxiety (B=.12, β=.19, p<0.01). |
|  | Weiss et al., 1995 | Emergency services personnel | There was a moderate correlation between social support and PTSD (r=-.25). |
|  | West et al., 2008 | Police | Being assaulted was a risk factor for PTSD (prevalence ratio 2.0, 95% CI 1.2, 3.5). |
| DURING- & POST-DISASTER: Professional support | Jenkins, 1996 | Emergency medical workers | Half of those who attended Critical Incident Stress Debriefing reported that it helped them cope (r=0.51, p<0.01). |
|  | Jenkins, 1997b | Fire dispatchers; 911 operators; police dispatchers | Those who received Critical Incident Stress Debriefing were significantly higher in avoidance (r(63)=0.32, p<0.01) 2.5 months post disaster, and had higher uninsured property loss estimates (r(44)=0.31, p<0.05). CISD did not account for any unique variance in any analyses when acute stress and coping skills were controlled for. |
|  | North et al., 2002a | Fire-fighters | 92% had participated in workplace diffusion/debriefings. Two thirds were satisfied with this & one third dissatisfied; however 89% said they would recommend the intervention. PTSD was not related to satisfaction or recommendation of the debriefings. However participants with other (non-PTSD) disorders were less likely to report satisfaction (50% vs 71%, X^2^=6.93, p<.01) or recommend them (82% vs 92%, X^2^=4.24, p<.05). |
|  | Tak et al., 2007 | Fire-fighters | Participation in a group counselling service was not associated with depressive symptoms. |
|  | Wang et al., 2011 | Military personnel | Pps who had not received psychological services during the rescue mission (chi square value=9.56, p=0.002) were more likely to have PTSD.  Multivariate analysis showed that the predictors of PTSD were earthquake experience score (odds ratio=6.46, 95% CI 4.47-9.32), **not receiving counselling** (odds ratio 3.28, 95 CI 1.31-8.20), and regular drinking (OR 2.42, 95% CI 1.04-5.62). |
| POST-DISASTER: Impact on life | Baum, 2014 | Social Workers | Changes in time & place of work correlated with PTG (0.25, p<0.05). PTG highly correlated with immersion in professional role (0.54, p<0.001) and moderately with role expansion (0.32, p<0.05). |
|  | Berninger et al., 2010a | Fire-fighters | Difficulty functioning at work afterwards was strongly associated with elevated PTSD risk (ORs ranged from 12.1 [95% CI 10.2-14.2] in year 1, to 23.0 [95% CI 14.6-36.3] in year 2) |
|  | Biggs et al., 2010 | Range of rescue personnel | All disaster workers with probable ASD reported high functional impairment while 22.4% of those without ASD reported high functional impairment (X^2^ = 29.94, p<0.001).  Disaster workers who reported functional impairment were 4.86 times more likely to have probable depression (Wald X^2^= 9.43, OR = 4.86, p< .05, CI = 1.77-13.33). |
|  | Bowler et al., 2012 | Police | Losing job after 9/11 was associated with high PCL scores (adjusted β= 4.45, SE=0.174, p<0.05). |
|  | Brackbill et al., 2009 | Rescue & officer workers | Job loss related to the event produced AORs for PTS symptoms ranging from 2.4 (95% CI, 1.9-3.1 [36.1% vs 11.7%]) among residents to 4.6 (95% CI, 4.0.-5.3 [56.4% vs 16.1%]) among rescue/recovery workers. |
|  | Corrigan et al., 2009 | Fire-fighters | Higher elevated PTSD scores were associated with functional job impairment and mental health–related medical leave (OR=2.69; 95% CI=2.33, 3.11; P<.001) and counselling service use (OR=2.53, CI 2.22-2.89, p<0.001). |
|  | Evans et al., 2006 | Disaster workers | Anger was significantly correlated with social functioning (r=0.41, p<0.001) but not occupational functioning. Social and occupational functioning were significantly correlated (r=0.50, p<0.001).  Social functioning significantly correlated with depression (r=0.35, p<0.01), distress (r=0.48, p<0.001), with disability (r=0.33, p<0.05), and PTSD (r=0.38, p<0.01).  Occupational functioning significantly correlated with depression (r=0.29, p<0.05), distress (r=0.32, p<0.01), and PTSD (r=0.26, p<0.05). |
|  | Evans et al., 2009 | Disaster workers | Significant correlation between impaired social/occupational functioning and PTSD symptoms (r=0.47, p<0.001). |
|  | Loganovsky et al., 2007 | Clean-up workers | For depressive disorder and PTSD the interaction effects of group X disorder in the analysis of variance tests were highly significant (p<0.001), indicating that clean-up workers with these two disorders had significantly greater work loss than either affected controls or non-affected individuals. |
|  | McFarlane, 1987 | Volunteer fire-fighters | Property loss was significantly correlated with GHQ score (p=0.000) and caseness on the GHQ (p=0.000). It was also the most significant loading variable in regression analysis, accounting for 4.9% of the variance (multiple r=0.23, adjusted r^2^=0.049, f=19.03, p=0.000).  Personal loss was not significantly correlated with GHQ score (p=0.2) or GHQ caseness (p=0.4) nor significant in regression analysis. |
|  | McFarlane, 1988b | Volunteer fire-fighters | The disordered (PTSD) and not-disordered groups were no different in their experience of the disaster as measured by losses. |
|  | McFarlane, 1988c | Volunteer fire-fighters | Property loss was the only disaster-related variable contributing significantly in contrast to the dominant role of three pre-disaster variables (neuroticism, avoidance, and history of psychological treatment). |
|  | McFarlane & Papay, 1992 | Volunteer fire-fighters | The PTSD-only group (X=1.2) suffered more property loss than the affective disorder, anxiety disorder, and no disorder groups (X=0.6, 0.8 and 1.0 respectively).  The PTSD only group had significantly greater property loss than either of the other 2 groups (F(2127)=3.9, p<.005).  Personal loss did not appear to affect mental health outcomes. |
|  | North et al., 2002a | Fire-fighters | Changes in relationships were more often reported by those with PTSD (42% vs 13%, p<.01), with positive changes equalling the negative. |
|  | Thormar et al., 2013 | Red Cross volunteers | Loss of resources (home, food/water, clothing or income) most influential exposure variable for depression (B=.20, β=.14) and predictive of PTSD (B=1.03, β=.16, p<0.05) and anxiety (B=.17, β=.19, p<0.001). |
|  | Thormar et al., 2014 | Red Cross volunteers | 62.2% had to spend the night outside their own home in the days following the earthquake. Mean PTSD scores were 24.0 for those who had to spend the night away from home and 20.9 for those who didn't; not significant. Subjective health complaints means were 20.2/19.4 respectively; not significant.  48.5% needed food/water aid. Mean PTSD scores for those who did/didn't were 25.7/20.2, p<.001. Mean subjective health complaints scores were 21.3/18.5, not significant.  22.2% needed clothes aid. Mean PTSD scores for those who did/didn't were 27.6/21.5, p<.01. Mean subjective health complaints scores were 24.1/18.7, p<.01.  28.6% needed shelter. Mean PTSD scores for those who did/didn't were 26.0/21.6, p<.01. Mean SHC scores were 21.3/19.2, not significant.  46.0% suffered financial difficulties because of the earthquake. Mean PTSD scores for those who did/didn't were 26.3/19.9, p<.001. Mean SHC scores were 22.3/17.8, p<.01.  39.3% needed financial assistance from others because of hardships caused by earthquake. Mean PTSD scores for those who did/didn't were 26.1/21.0, p<.001. Mean SHC scores were 22.1/18.5, p<.05. |
|  | VanDevanter et al., 2014 | Nurses | Deployed nurses who also experienced **personal storm-related loss, damage** or injury were no more likely to report deployment-related stress than nurses who did not experience a personal storm-related event. |
|  | West et al., 2008 | Police | Risk factors associated with depression included rare family contact, uninhabitable home, and isolation from the NOPD (PR (95% CI) = 1.6 (1.2, 2.1); 1.4 (1.02, 1.8); 1.5 (1.1, 2.0) respectively). |
| POST-DISASTER: Life events | McFarlane, 1987 | Volunteer fire-fighters | Recent life events were significantly correlated with GHQ score (p=0.000) and caseness on the GHQ (p=0.000). This was also a significant loading variable in regression analysis, accounting for 3.8% of the variance (multiple r=0.30, adjusted r^2^=0.087, f=17.69, p=0.000). |
|  | McFarlane, 1988c | Volunteer fire-fighters | At 29 months, exposure to subsequent fires did not differentiate the outcome of any of the groups. |
|  | McFarlane & Papay, 1992 | Volunteer fire-fighters | The affective disorder group suffered significantly more adversity in the aftermath of the disaster (X=2.1) than the PTSD only and not-disordered groups (X=1.0 and 1.0 respectively; F(3145)=4.5; p<.01).  The group who had PTSD as well as some other disorder were more likely than the no disorder group to have experienced adversity both before the disaster (F(2127=4.2, p<.05) and after (F(2127)=6.0, p<.01). |
|  | Pietrzak et al., 2012 | Police | Associated with full // subsyndromal PTSD respectively:  Number of life stressors since 9/11 (1.25 (95% CI 1.14-1.36 // 1.22, 95% CI 1.14-1.29) |
|  | Witteveen et al., 2007 | Fire-fighters, police | In fire-fighters, number of post-disaster life events was associated with all SRIP scales, SCL-90-R anxiety and depression (all B=1.02).  In police, number of post-disaster life events was associated with SRIP total, SRIP intrusion, SRIP avoidance, SRIP hyperarousal, anxiety, depression, somatic complaints (B=1.02 for all), fatigue (B=1.04) and IES total (B=1.09). |
| POST-DISASTER: Media | Jenkins, 1997a | Emergency workers | Watching TV was significantly positively correlated with anxiety post disaster (r=0.38, p<0.05). |
|  | McFarlane, 1988c | Volunteer fire-fighters | The persistent delayed-onset (X^2^=32.79, df 3, p=.000), persistent chronic (X^2^=2.65, df 3, p=.000) and resolved chronic (X^2^=15.67, df 3, p=.003) groups were all significantly more distressed by television reminders of the fire. |
|  | Nishi et al., 2012 | DMAT workers | Watching TV for more than 4 hours per day at 1 month post-earthquake was predictive of PTSD symptoms (univariate regression: β=10.3, 95% CI 5.02, 15.5. multivariate regression: β=5.24, 95% CI 0.27, 10.2). |
|  | Warren et al., 2003 | Emergency medicine physicians | Watching over 3 hours of daily media coverage was not associated with more emotional distress (t(106)=0.24, p=.81). |
| POST-DISASTER: Coping strategies | Bhushan et al., 2012 | NGO Relief Volunteers | Proactive coping positively correlated with relating to others (r=0.692, p<.01), new possibilities (r=0.594, p<.01), personal strength (r=0.596, p<.01), spiritual change (r=0.598, p<.01), and the total posttraumatic growth score (r=0.681, p<.01). |
|  | Brown et al., 2002 | Fire-fighters | For those in the low incident frequency group, higher GHQ scores were associated with higher scores on locus of control, indicating greater externality, lower scores on emotion-focused coping, and higher scores on avoidance, R^2^=0.43, F(5, 123) = 18.36, p<0.001.  For those in the high incident frequency group, higher GHQ scores were associated with negative emotions, lower scores on task-focused coping, and higher scores on avoidance, R^2^ =0.32, F(5, 114) = 10.88, p<.001.  Avoidance coping accounted for most of the explained variance in psychological distress. |
|  | Chang et al., 2003 | Fire-fighters | Multivariate logistic regression indicated:  Confrontive coping was a significant predictor of psychiatric morbidity (OR 1.95, 95% CI 130-2.92, p<0.01).  Distancing (OR 2.20, 95% CI 1.33-3.64, p<0.01), escape-avoidance (OR 1.43, 95% CI 1.03-1.98, p<0.05), and positive reappraisal (OR 0.59, 95% CI 0.38-0.90, p<0.05) were significant predictors of posttraumatic morbidity. |
|  | Chang et al., 2008 | Fire-fighters | All coping strategies were significantly positively correlated with both general psychiatric morbidity and post-traumatic morbidity (p<0.001). Confrontive coping (β=-0.68, SE=0.29, p<0.05), distancing (β =-0.6, SE=0.26, p<0.05), and planned problem-solving (β=-0.54, SE=0.25, p<0.05) strategies significantly reduced the effect of direct rescue effort involvement on general psychiatric morbidity. |
|  | Eidelson et al., 2003 | Psychologists | Positive feelings were significantly positively associated with volunteer activities (β =0.19, p<0.001), a changed view on irrational fear (β =0.09, p<0.05), & personal life affected (β =0.13, p<0.01). |
|  | Hodgkinson & Shepherd, 1994 | Social Workers | **39% of the variance in psychological wellbeing could be explained by variables reflecting coping style (hardiness, p<0.01),** prior life events (p<0.001) & role difficulties/contact with client's distress (identification p<0.05; 88% reported ruminating about client's experiences). |
|  | Jenkins, 1997a | Emergency workers | Social-emotional support was strongly positively correlated with treatment seeking (r=0.67, p<0.001), cognitive behavioural approach (r=0.62, p<0.001) & social approach (r=0.43, p<0.01). Spirituality was strongly positively correlated with situation avoidance (r=0.6, p<0.001), treatment seeking (r=0.5, p<0.01) & event rumination (r=0.51, p<0.01).  Asserting responsibility was not significantly correlated to any stress response. Of 54 possible inter-correlations between coping & activities, only 2 were significant; - spirituality & thinking about the incident (r(26)=0.43, p<0.05) and distress from the media (r(31)=0.5, p<0.01). Stress & routine activities inter-correlations - treatment seeking was related to creative activities (r(26=0.37, p<0.05), those who had a more cognitive behavioural approach to coping were more distressed by the media (r(32)=0.4, p<0.05).  The most common correlates of acute distress were Spirituality (which was especially strongly related to psychosomatic symptom increases) and Self-isolation/Withdrawal. Event rumination was significantly positively associated with Obsessive/compulsive scale post-disaster (r=0.4, p<0.05) but significantly negatively associated at FU (r=-0.37, p<0.05).  Thinking about the incident had similar delayed effect with obsessive/compulsive (r=0.44, p<0.05 & -0.39, p<0.05) and anxiety (r=0.53, p<0.05 & -0.36, p<0.05). Exercise helped with hostility (r=-0.54, p<0.05) and obsessive/compulsive (r=-0.34, p<0.05) at FU, delayed improvement. |
|  | Jenkins, 1997b | Fire dispatchers; 911 operators; police dispatchers | Planned effort was significantly correlated with positive reappraisal (r=0.47, p<0.001) and social support (r=0.57, p<0.001). Anger was significantly correlated with positive reappraisal (r=0.42, p<0.001).  In multiple regression analyses, distancing and anger explained 7% and 6% of variance in avoidance; positive reappraisal explained 8% of psychosomatic symptom variance. |
|  | Linley & Joseph 2006 | Disaster Response Workers | Approaching acceptance in attitudes towards death was associated with PTG at FU only (r=0.55, p<0.01).  IES avoidance (r=0.56, p<0.001), fear of death (r=0.41, p<0.01), avoiding thinking about death (r=0.68, p<0.001 & r=0.4, p<0.01) were associated with negative changes. PTG, positive & negative changes appear stable over time, with test retest correlation >0.5 over 6 month FU. |
|  | McFarlane, 1988b | Volunteer fire-fighters | Results suggested that intrusive imagery and avoidant thoughts following low exposure tended to predict PTSD more than intrusive imagery and avoidant thoughts after intense exposure. |
|  | Soffer et al., 2011 | Rescue workers | Only crisis of meaning (p<.01) and negative affect (p<.001) were associated with dissociation and PTSD. |
|  | Tucker et al., 2002 | Red Cross volunteers | The most frequently reported means of coping were spending time with others and focusing on the positive. There were no significant differences between those who had used/not used each coping technique on mean difference in posttraumatic stress, difference in depression, change in alcohol use or seeking mental health treatment. |
|  | Zhen et al., 2012 | Red Cross nurses | Traumatic stress symptoms predicted by **avoidance of traumatic thoughts during earthquake** (stats not reported). |

Table S3. Summary of results

| **Theme / risk factor** | **Studies (n)** | **Quality (poor / adequate / good)*** | **Findings** |
| --- | --- | --- | --- |
| PRE-DISASTER: Occupational factors | 22 | 0 / 14 / 8 | 4 studies showed that workers in different roles had different outcomes. 3 studies reported that professionals had better outcomes than volunteers while 1 study reported that volunteers had better outcomes. 1 study reported inconsistent results regarding the relationship between professional status and outcomes.  4 studies reported that longer time in employment was associated with better outcomes. 3 studies reported that longer time in employment was associated with poorer outcomes. 4 studies reported no significant effect.  1 study reported that job dissatisfaction was associated with poorer outcomes. 1 study showed that job dissatisfaction was associated with alcohol misuse but not PTSD. |
| PRE-DISASTER: Specialised training and preparedness | 18 | 0 / 9 / 9 | 5 studies showed that training was associated with better outcomes. 1 study showed no significant effect.  3 studies reported that sense of preparedness/competence was associated with better outcomes. 0 studies showed no effect.  2 studies showed that previous disaster experience was associated with better outcomes. 1 study reported that previous disaster experience was associated with poorer outcomes. 5 studies reported no significant effect. 1 study showed a significant effect of previous disaster experience in univariate but not multivariate analysis. |
| PRE-DISASTER: Life events and health | 22 | 1 / 9 / 12 | 15 studies reported that major life events/life stress prior to the disaster were associated with poorer outcomes post-disaster. 0 studies showed no significant effect.  9 studies reported that history of psychiatric disorder were associated with poorer outcomes post-disaster. 0 studies showed no significant effect. |
| DURING DISASTER: Exposure | 50 | 1 / 22 / 27 | 41 studies reported that greater exposure was associated with greater psychological distress. 7 studies reported no significant relationship between exposure and outcomes. 1 study showed inconsistent results at different time-points. 1 study showed a significant effect of exposure in univariate but not multivariate analysis.  In particular those working in close proximity to the epicentre of the disaster tended to have worse outcomes, as did those dealing with dead bodies/injuries. |
| DURING DISASTER: Duration on site and arrival time | 21 | 0 / 11 / 10 | 11 studies reported that longer duration on site was associated with poorer outcomes. 5 studies reported no significant relationship between duration on site and wellbeing.  7 studies reported that early arrival time at the site was associated with poorer outcomes. 1 study reported that early arrival time was associated with better outcomes. 3 studies showed no significant relationship between arrival time and wellbeing. |
| DURING DISASTER: Emotional involvement | 4 | 0 / 2 / 2 | 4 studies reported that over-identifying with victims or survivors was associated with worse outcomes. 0 studies showed no significant effect. |
| DURING DISASTER: Peri-traumatic distress / dissociation | 10 | 0 / 4 / 6 | 8 studies reported that experiencing particularly stressful situations during the disaster and/or responding to them by dissociating were associated with poorer wellbeing. 2 studies reported no significant relationship between peri-traumatic distress or dissociation and wellbeing. |
| DURING DISASTER: Role-related stressors | 15 | 0 / 3 / 12 | 14 studies reported that role-related factors negatively affected outcomes. These factors included role ambiguity, tasks outside of usual remit, lack of control over work, heavy workload and difficult tasks. 1 study showed no relationship between a role-related factor (caseload) and wellbeing. |
| DURING DISASTER: Perceptions of safety, threat and risk | 10 | 0 / 7 / 3 | 8 studies reported that feeling at risk/unsafe was associated with poorer outcomes. 1 study reported no significant relationship between perception of safety and wellbeing. 1 study showed a significant effect of the perception of safety in univariate but not multivariate analysis. |
| DURING DISASTER: Harm to self or close others | 20 | 0 / 9 / 11 | 5 studies reported that sustaining an injury or having a near-death experience were associated with poorer outcomes. 1 study showed no significant relationship between personal injury and outcomes. 2 studies showed inconsistent results (e.g. results were different for different groups of participants).  8 studies reported that having close others injured or killed was associated with poorer outcomes. 1 study showed no significant relationship between harm to close others and wellbeing. 1 study showed a significant effect of harm to others in univariate but not multivariate analysis. |
| DURING DISASTER: Social support | 26 | 2 / 10 / 14 | 20 studies reported that social support was associated with outcomes (i.e., the greater the support, the better the outcomes). 4 studies showed no significant relationship between social support and wellbeing. 2 studies reported inconsistent results. 2 studies reported that negative social experiences such as harassment were associated with worse outcomes.  Support from managers/colleagues appeared to be particularly beneficial. |
| POST-DISASTER: Professional support | 5 | 1 / 2 / 2 | 2 studies reported a positive effect of professional support. 3 studies showed no significant relationship between professional support and wellbeing. |
| POST-DISASTER: Impact on life | 18 | 0 / 6 / 12 | 11 studies reported that impact on personal life (such as needing food/clothing aid or shelter; property loss) or professional life was associated with negative outcomes. 1 study reported that impact on life was associated with post-traumatic growth. 2 studies showed no significant relationship between impact on life and wellbeing. 4 studies showed inconsistent results. |
| POST-DISASTER: Life events | 5 | 0 / 1 / 4 | 4 studies reported that experiencing stressful life events post-disaster was associated with poorer outcomes. 1 study showed no significant relationship between post-disaster life events and wellbeing. |
| POST-DISASTER: Media | 4 | 1 / 1 / 2 | 3 studies reported that media exposure was associated with poorer outcomes. 1 study showed no significant relationship between media exposure and wellbeing. |
| POST-DISASTER: Coping strategies | 13 | 1 / 8 / 4 | 12 studies reported that coping style was associated with outcomes. 1 study showed no significant relationship between coping style and wellbeing.  Avoidance and distancing tended to be associated with poorer outcomes, while proactive coping, acceptance and positive thinking tended to be associated with better outcomes. |

*Poor quality=<50%; adequate quality=51-80%; good quality=>81%
